# Lipodystrophy in methylmalonic acidemia associated with elevated FGF21 and abnormal methylmalonylation

**DOI:** 10.1172/jci.insight.174097

**Published:** 2024-02-22

**Authors:** Irini Manoli, Justin R. Sysol, PamelaSara E. Head, Madeline W. Epping, Oksana Gavrilova, Melissa K. Crocker, Jennifer L. Sloan, Stefanos A. Koutsoukos, Cindy Wang, Yiouli P. Ktena, Sophia Mendelson, Alexandra R. Pass, Patricia M. Zerfas, Victoria Hoffmann, Hilary J. Vernon, Laura A. Fletcher, James C. Reynolds, Maria G. Tsokos, Constantine A. Stratakis, Stephan D. Voss, Kong Y. Chen, Rebecca J. Brown, Ada Hamosh, Gerard T. Berry, Xiaoyuan Shawn Chen, Jack A. Yanovski, Charles P. Venditti

**Affiliations:** 1 Metabolic Medicine Branch, National Human Genome Research Institute;; 2 Mouse Metabolism Core, National Institute of Diabetes and Digestive and Kidney Diseases;; 3 Section on Growth and Obesity, Eunice Kennedy Shriver National Institute of Child Health and Human Development; and; 4 Office of Research Services, Division of Veterinary Resources, NIH, Bethesda, Maryland, USA.; 5 Department of Genetic Medicine, Johns Hopkins University, Baltimore, Maryland, USA.; 6 Diabetes, Endocrinology, and Obesity Branch, National Institute of Diabetes and Digestive and Kidney Diseases;; 7 Radiology and Imaging Sciences Department, Clinical Center;; 8 Ultrastructural Pathology Section, Center for Cancer Research; and; 9 Section on Endocrinology & Genetics, Eunice Kennedy Shriver National Institute of Child Health and Human Development, NIH, Bethesda, Maryland, USA.; 10 Department of Radiology, Boston Children’s Hospital, Harvard Medical School, Boston, Massachusetts, USA.; 11 Division of Genetics and Genomics, The Manton Center for Orphan Disease Research, Boston Children’s Hospital, Boston, Massachusetts, USA.; 12 Department of Pediatrics, Harvard Medical School, Boston, Massachusetts, USA.; 13 Laboratory of Molecular Imaging and Nanomedicine, National Institute of Biomedical Imaging and Bioengineering, NIH, Bethesda, Maryland, USA.

**Keywords:** Genetics, Therapeutics, Amino acid metabolism, Mouse models, Obesity

## Abstract

A distinct adipose tissue distribution pattern was observed in patients with methylmalonyl-CoA mutase deficiency, an inborn error of branched-chain amino acid (BCAA) metabolism, characterized by centripetal obesity with proximal upper and lower extremity fat deposition and paucity of visceral fat, that resembles familial multiple lipomatosis syndrome. To explore brown and white fat physiology in methylmalonic acidemia (MMA), body composition, adipokines, and inflammatory markers were assessed in 46 patients with MMA and 99 matched controls. Fibroblast growth factor 21 levels were associated with acyl-CoA accretion, aberrant methylmalonylation in adipose tissue, and an attenuated inflammatory cytokine profile. In parallel, brown and white fat were examined in a liver-specific transgenic MMA mouse model (*Mmut*^–/–^ Tg^INS-Alb-Mmut^). The MMA mice exhibited abnormal nonshivering thermogenesis with whitened brown fat and had an ineffective transcriptional response to cold stress. Treatment of the MMA mice with bezafibrates led to clinical improvement with beiging of subcutaneous fat depots, which resembled the distribution seen in the patients. These studies defined what we believe to be a novel lipodystrophy phenotype in patients with defects in the terminal steps of BCAA oxidation and demonstrated that beiging of subcutaneous adipose tissue in MMA could readily be induced with small molecules.

## Abstract 2

<p>Subcutaneous adipose tissue expansion with beige fat properties is observed in methylmalonic acidemia associated with aberrant methylmalonylation and high FGF21.</p>

## Graphical abstract

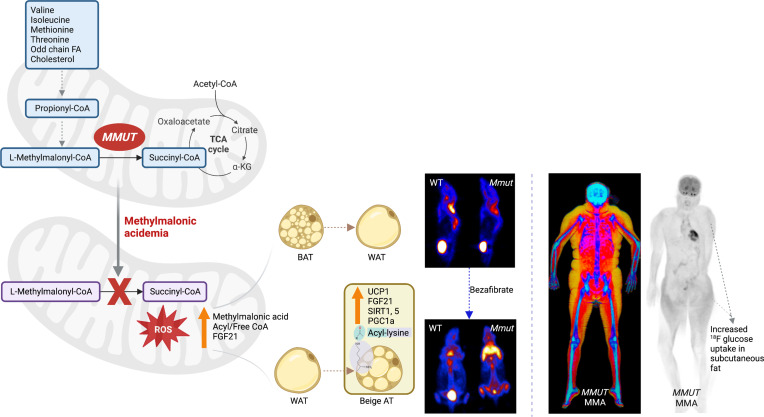

## Introduction

Large-scale genomic and metabolomic studies have demonstrated a key role for branched-chain amino acid (BCAA) metabolism in obesity, insulin resistance, and type 2 diabetes mellitus (T2DM) (1–4), with a low-protein/low-BCAA diet promoted as a means to improve metabolic health (5, 6). While leucine has well-defined metabolic effects on insulin secretion, skeletal muscle anabolism, and hypothalamic appetite circuits (7, 8), the roles of valine and isoleucine oxidative pathways in fat physiology and the metabolic syndrome remain relatively unexplored (9, 10). Levels of propionyl- (C3), and methylmalonyl- or succinylcarnitine (C4DC), derived from valine and isoleucine oxidation, correlate with impaired glycemic control and T2DM (11) whereas succinate accumulation averts diet-induced obesity by stimulating brown fat thermogenesis (12). Despite this accumulating evidence, focused studies on glucose and lipid metabolism in patients or murine models of inborn errors affecting BCAA oxidative pathways, typically associated with extreme BCAA metabolite perturbations, remain limited (9, 13–15).

Methylmalonic acidemia (MMA, OMIM #251000) is a prototypic organic aciduria caused by impaired activity of the 5′deoxy-adenosylcobalamin–dependent (vitamin B_12_–dependent) mitochondrial enzyme methylmalonyl-CoA mutase (MMUT) (MMUT or MCM, EC 5.4.99.2) (16). MMUT catalyzes the isomerization of l-methylmalonyl-CoA to succinyl-CoA at the terminal step of propionyl-CoA metabolism. Pathogenic variants in *MMUT* result in supraphysiologic concentrations of methylmalonic acid and C3- and C4DC-derived toxic metabolites in bodily fluids and tissues. Cell-autonomous mitochondrial dysfunction and abnormal mitophagy are the hallmarks of disease pathophysiology (15, 17–19). Affected organs display megamitochondria, while aberrant posttranslational modifications (PTMs), including methylmalonylation of lysine residues, disturb the function of several critical mitochondrial enzymes (20). Although patients with MMA are maintained on high-fat and -carbohydrate diets because of lifelong protein restriction and develop increased fat mass and obesity (21–24), insulin resistance and T2DM are not among the recognized chronic disease complications (14, 15, 25, 26). Moreover, mice and patients exhibit extreme elevations of fibroblast growth factor 21 (FGF21) (15, 27), a key metabolic regulator with pleiotropic effects in liver and adipose tissue (28, 29) that has been associated with resistance to obesity, improved glucose tolerance, and reduced fat accumulation and inflammation in the liver, making it a promising therapeutic target for obesity, nonalcoholic steatohepatitis, and cardiometabolic phenotypes (30–32).

In the present study, we describe a distinct lipodystrophy phenotype in children and young adults with MMA enrolled in a natural history protocol (ClinicalTrials.gov ID: NCT00078078) and further explore white and brown adipose tissue (WAT and BAT) physiology in a mouse model of MMA that relies upon a germ line transgene to express MMUT in a hepatocyte-restricted fashion (Tg^INS-Alb-Mmut^) that overcomes the neonatal lethal phenotype of the knockout mice and allows studies aimed at dissecting the molecular mechanisms underlying the response to environmental and dietary stress, a main cause for the high morbidity and mortality in this disorder (18). The muscle-transgenic (Tg^INS-MCK-Mmut^) mice presented previously are fragile and require high-fat and -carbohydrate diet and heating pads for survival, which would compromise studies on adipose tissue physiology that were the focus of the current work (15). Bezafibrates were administered to restore mitochondrial biogenesis and activate peroxisome proliferator–activated receptor (PPAR) targets in brown and white fat in the *Mmut*^–/–^ Tg^INS-Alb-Mmut^ mice, which resulted in improved survival, restoration of BAT pathology, and beiging of subcutaneous fat depots that then resembled the fat distribution seen in the patients with MMA. In aggregate, studies in patients and mice with MMA revealed a subcutaneous adipose tissue depot with beige fat properties, which was associated with high circulating FGF21 and an attenuated inflammatory cytokine profile, highlighting the role of disrupted valine and isoleucine oxidation and resulting PTMs in adipose tissue physiology and suggesting new therapeutic targets for the disease.

## Results

### Clinical characterization of the lipodystrophy observed in the MMA patient cohort.

**Figure 1:**
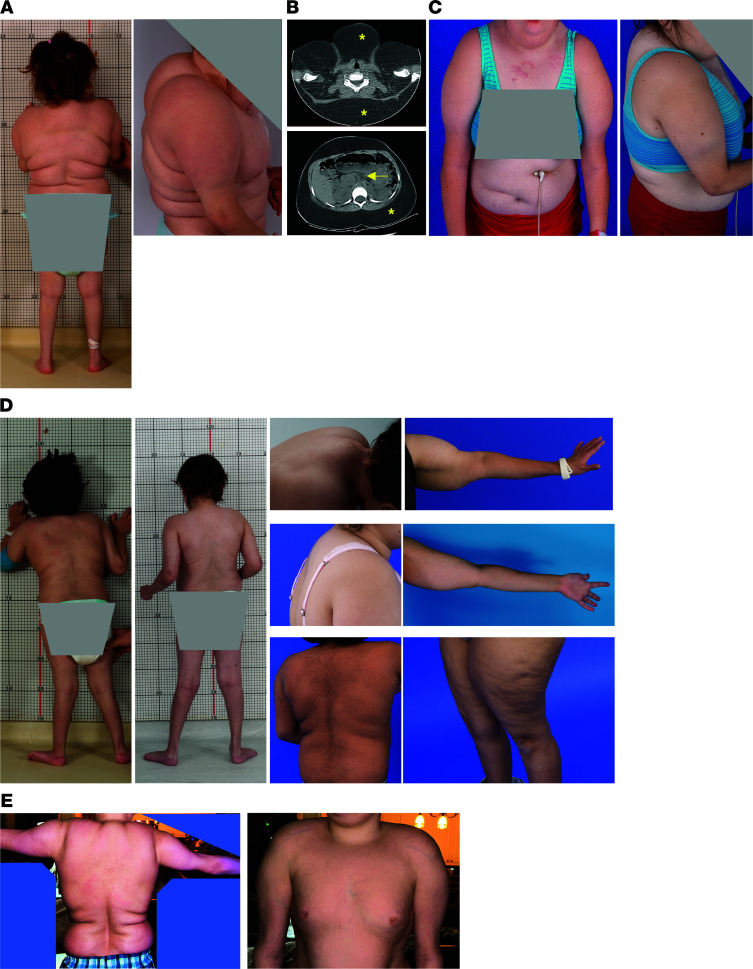
Subcutaneous fat accumulation with lipodystrophy of lower extremities in MMA. (**A**) Cushingoid phenotype of the index patient, a 6-year-old White girl, with fat accumulation in the torso, dorsocervical area, and shoulders/upper arms, with lipodystrophy of the distal arms and legs. (**B**) Axial sections of cervical and abdominal CT imaging demonstrating accumulation of subcutaneous fat, marked with asterisks. Arrow points to the minimal visceral fat present in the abdominal CT section. (**C**) Index patient at 18 years of age showing subcutaneous fat depots over her torso and upper arms. (**D**) A similar fat accumulation in proximal upper and lower limbs with wasting in distal extremities and presence of dorsocervical hump in 7 additional male and female patients with *mut^0^* (low to no methylmalonyl-CoA enzymatic activity) or *MMUT*-type and (**E**) 1 *cblB*- or *MMAB*-type MMA of various racial and ethnic backgrounds (White, Asian, Pacific Islander, and Hispanic).

The index patient, a 6-year-old girl with severe MMUT deficiency, had developed a pronounced abnormal body habitus, with massive accumulation of adipose tissue in her torso, dorsocervical area, and upper arms and legs, with wasting of fat and muscle in the distal extremities (Figure 1A) over 2 years before her evaluation. Notably, the increased abdominal fat was almost exclusively localized in the subcutaneous depot, with minimal visceral fat (Figure 1B). Endocrine and imaging studies ruled out Cushing’s syndrome (see Supplemental Methods for additional clinical information; supplemental material available online with this article; https://doi.org/10.1172/jci.insight.174097DS1). Genetic testing, including comparative genomic hybridization microarray to rule out copy number variation, mitochondrial DNA sequencing, and next-generation sequencing of a panel of 29 congenital and acquired lipodystrophy genes (*ADRA2A*, *AGPAT2*, *AKT2*, *ATP6V0A2*, *BANF1*, *BSCL2*, *CAV1*, *CAVIN1*, *CIDEC*, *FBN1*, *IGF1R*, *KCNJ6*, *LIPE*, *LMNA*, *LMNB2*, *MFN2*, *PCYT1A*, *PIK3R1*, *PLIN1*, *POLD1*, *PPARG*, *PSMA3*, *PSMB4*, *PSMB8*, *PSMB9*, *SPRTN*, *TBC1D4*, *WRN*, *ZMPSTE24*), including mitofusin-2 (*MFN2*) associated with multiple lipomatosis, was unrevealing for additional diagnoses beyond *MMUT* MMA. The patient developed chronic renal disease and received a combined liver and kidney transplant at age 18. She developed diabetes requiring insulin treatment after the transplantation procedure, despite only a brief course of steroids for immunosuppression, with no notable change in the lipodystrophy phenotype after approximately 2 years from the surgery (Figure 1C). The plasma FGF21 concentration dropped from 7,956 pg/mL to 33.8 (normal range 71.47 ± 102.5 pg/mL) after transplant, consistent with our previous observations (15).

**Figure 2:**
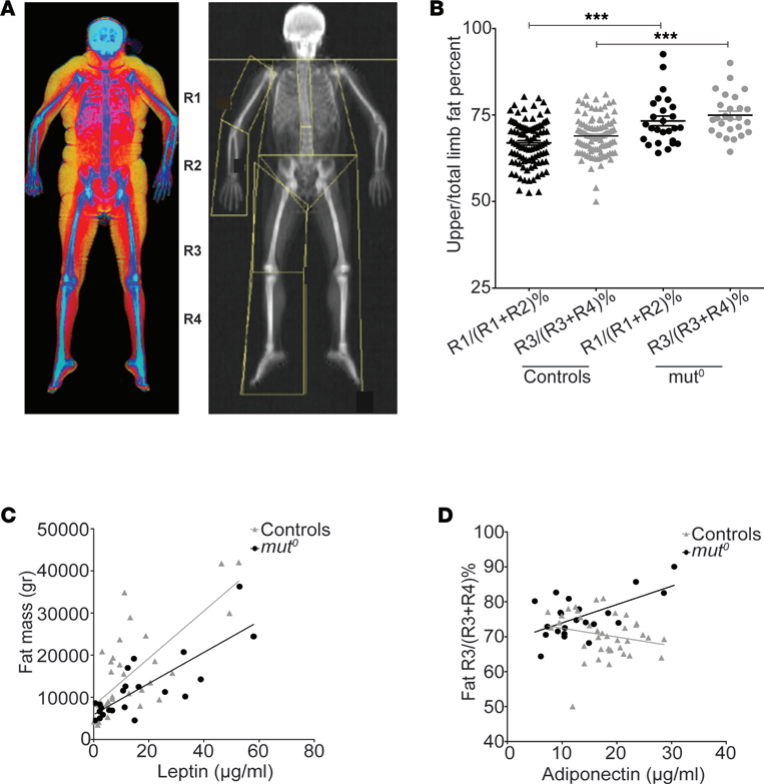
Appendicular segmental DXA analysis and serum adipokines suggest distinct metabolic properties of the subcutaneous fat depot in participants with MMA. (**A**) Bone densitometry demonstrating subcutaneous fat accumulation (yellow) in the color image and the appendicular segments used for fat and fat-free mass analysis: upper and lower arm (R1 and R2) and upper and lower leg (R3 and R4), respectively. (**B**) The ratio of proximal to total limb fat mass for upper (R1/R1+R2) and lower extremities (R3/R3+R4) was generated, as a metric of the phenotypic severity. The fat accumulation in the proximal extremity segment was significantly higher in the *mut^0^* MMA patient cohort as opposed to matched controls for both upper and lower extremities. Data represent the mean ± SEM. ****P < 0.0001, by 1-way ANOVA with Bonferroni’s post hoc test. (**C**) Plasma leptin concentrations correlated with fat mass (FM) in both the *mut^0^* MMA (*r* = 0.806, *P* < 0.0001, *R*^2^ = 0.650) and the control (*r* = 0.738, *P* < 0.0001, *R*^2^ = 0.545) groups. (**D**) The ratio of proximal/total lower extremity FM% correlated positively with plasma adiponectin concentrations in patients with *mut^0^* MMA (*r* = 0.601, *P* = 0.0031, *R*^2^ = 0.360) but not in the controls (*r* = –0.21, *P* = 0.19, *R*^2^ = 0.045).

We subsequently identified similar, albeit less extreme, phenotypes in additional patients with *mut^0^* (low to no methylmalonyl-CoA enzymatic activity) or *MMUT*-type and *cblB*- or *MMAB*-type MMA (Figure 1, D and E, respectively), of either sex and from various ethnic backgrounds and ages. To generate a metric that would objectively define the extent of abnormal fat distribution, we used subregion analysis of whole-body composition dual-energy x-ray absorptiometry (DXA) studies, as previously described (33, 34). We measured the fat content of different appendicular segments and calculated the percentage proximal/total extremity [R1/(R1+R2)% and R3/(R3+R4)%] fat mass (Figure 2A). Forty-six patients with MMA (28 male/18 female, 32 *mut/MMUT*, 8 *cblA/MMAA*, and 6 *cblB/MMAB* subtype) and 99 age-, sex-, race- and ethnicity-, and BMI-matched controls (61 male, 38 female) ascertained from an NIH pediatric obesity cohort were evaluated. Patients with MMA had increased subtotal (total body minus head) fat mass percentage (FM%) compared with controls (30.9% ± 10.5% vs. 26.5% ± 9.8%, mean ± SD, *P* = 0.0147; 31.7% ± 10.4%, *P* = 0.0061 for *mut^0^* subgroup; unpaired 2-tailed *t* test for all), especially girls (37.9 ± 7.5% vs. 26.5 ± 9.8%, *P* = 0.009, Supplemental Figure 1, A and B). Segmental analysis showed increased proximal/total upper and lower extremity FM% in patients with MMA compared with matched controls [73.2% ± 6.6% vs. 66.9% ± 6.4% for upper extremities, *P* < 0.0001; 74.3% ± 5.7% vs. 68.9% ± 5.6%, *P* < 0.0001 for lower extremities; data shown for *mut^0^*; *N* = 26 (Figure 2B and Table 1)].

We next compared concentrations of 2 serum adipokines, leptin and adiponectin (35–38), and their correlations with fat indices in patients with MMA and matched controls. In our study cohorts, plasma leptin concentrations were positively correlated with FM% in both the MMA and the control groups, as expected (Figure 2C), while adiponectin concentrations showed a negative correlation with FM% only in controls (Supplemental Figure 1C). Furthermore, the ratio of proximal/total FM% correlated positively with serum adiponectin concentrations in patients with *mut^0^* MMA, but not in controls, supporting the hypothesis that subcutaneous fat properties in the MMA patient cohort are different from those of controls (for lower extremity: *r* = 0.601, *P* = 0.0031, *R*^2^ = 0.360, compared with *r* = –0.21, *P* = 0.19, *R*^2^ = 0.045 in the controls; Figure 2D; upper extremity correlations are depicted in Supplemental Figure 1D). The unexpected correlation of the subcutaneous fat accumulation in proximal limb regions with adiponectin concentrations prompted further studies in MMA animal models and in the blood and tissue samples from our patients, starting with the index patient (Supplemental Figure 1, E–G), and healthy controls.

### Exploration of adipose tissue pathophysiology in Tg Mmut^–/–^ Tg^INS-Alb-Mmut^ mice at baseline and after cold exposure.

Tg MMA murine models that express either liver-restricted (*Mmut^–/–^* Tg^INS-Alb-Mmut^) (18) or muscle-restricted (*Mmut^–/–^* Tg^INS-MCK-Mmut^) (15) rescue transgenes manifested cold intolerance and appeared to shiver at the regular mouse facility room temperature of 24°C, which is below the thermoneutral environment for mice of 30°C, requiring housing on heating pads. This suggested a defect of nonshivering thermogenesis, the main function of BAT in rodents (39, 40). We chose the transgenic liver-restricted *Mmut^–/–^* Tg^INS-Alb-Mmut^ murine model that was previously shown to replicate characteristic extrahepatic manifestations of the MMA phenotype (18) to investigate BAT and WAT pathophysiology.

**Figure 3:**
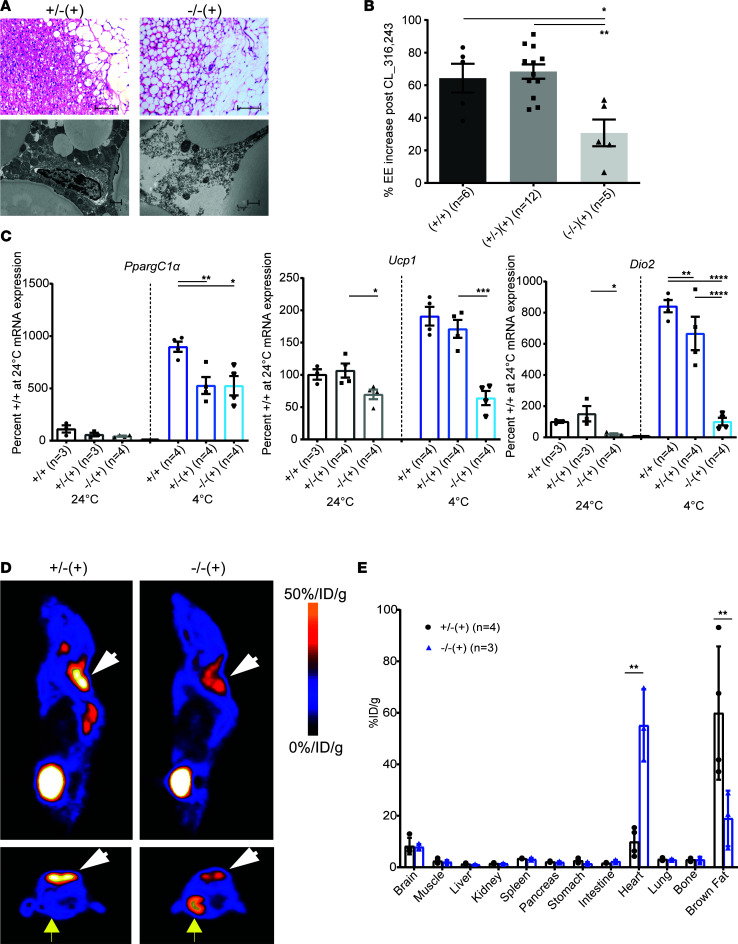
MMA mice display cold intolerance associated with abnormal brown fat pathology and function. (**A**) Sets of 4- to 5-month-old mutant mice, *Mmut*^–/–^ Tg^INS-Alb-Mmut^, depicted as (–/–) (+), and heterozygous littermates (+/–) (+) exposed to high-protein diet for 2 months were euthanized and their interscapular brown adipose tissue (BAT) sectioned and H&E-stained. Mutant mice display markedly abnormal BAT with decreased cellular content (fewer nuclei) and large lipid droplets as opposed to controls (scale bars: 100 μm). Ultrastructural studies demonstrated multinodular lipid droplets and numerous mitochondria with normal cristae in BAT from heterozygous mice, as opposed to large lipid lobules and sparse mitochondria with abnormal density and cristae in the mutant animals (scale bars: 1 μm). (**B**) Effects of acute β_3_-adrenergic stimulation on energy expenditure. *Mmut*^–/–^ Tg^INS-Alb-Mmut^ showed a 50% lower increase in their metabolic rate in response to a single dose of β-agonist, CL-316,243, compared with their control littermates (*P* = 0.0016, 1-way ANOVA with Bonferroni correction). (**C**) Quantitative RT-PCR results showing fold-change mRNA expression for *PpargC-1α*, *Ucp1*, and *Dio2* at room temperature and after cold exposure (4°C for 3 hours). *PpargC-1α* showed increased transcription after cold exposure, albeit to a lesser degree than in WT animals in both mutant and heterozygous mice, while *Ucp1* and *Dio2* had lower expression at baseline and showed no to little change with cold stress. (**D**) Sagittal and transaxial micro-PET images of mice, reared on high-protein chow for 2 months, after injection with ^18^F-fluoro-d-glucose (^18^F-FDG). White arrowheads point to the interscapular BAT. Mutant mice had significantly decreased uptake in the BAT, while they showed high uptake in the heart tissue (yellow arrows, bottom panels), compared with heterozygotes. %ID/g, injected dose%/gram. (**E**) Biodistribution of radioactive label in various tissues. *P < 0.01, **P < 0.001, ***P < 0.0001, and ****P < 0.00001.

*Mmut^–/–^* Tg^INS-Alb-Mmut^ mice were fed a high-protein (HP) diet as previously described (18) with subsequent harvesting of adipose tissue. The BAT in the mutant animals, dissected by a veterinary pathologist, resembled the appearance of WAT, with large, monovesicular, lipid-laden adipocytes and decreased nuclear density compared with heterozygous control littermates (Figure 3A). Furthermore, electron microscopy showed strikingly abnormal ultrastructure in BAT of mutant animals, with sparse, small mitochondria with abnormal cristae, and large fat lobules, compared with the numerous mitochondria and microvesicular fat droplets observed in the heterozygous animals (Figure 3A).

We subsequently housed the mice individually in temperature-controlled metabolic chambers and studied body composition, energy expenditure, and ambulatory activity. Experiments were performed at room temperature (24°C) and at thermoneutral temperature for mice (30°C), as well as after injection of the selective β_3_-adrenergic receptor (*β_3_*-AR) agonist, CL-316,243 (41). Stimulation of *β_3_*-ARs causes lipolysis in WAT and thermogenesis in BAT, with an associated increase in energy expenditure, and can be used to test BAT function. *Mmut^–/–^* Tg^INS-Alb-Mmut^ mice had lower FFM (or lean mass), lower quadriceps, spleen, and liver weights; lower inguinal (WAT) mass; and increased BAT mass compared with wild-type or heterozygous littermates (Supplemental Figure 2, A–C). They also exhibited lower oxygen consumption per gram of FFM (Supplemental Figure 2D) that was associated with decreased ambulatory activity (Supplemental Figure 2E). These differences were significant at room temperature (*P* = 0.005 and 0.02, respectively) but not at 30°C. Respiratory exchange ratios were not different between wild-type, heterozygous, and mutant mice (data not shown).

When injected with the *β_3_*-AR agonist (CL-316,243), the *Mmut^–/–^* Tg^INS-Alb-Mmut^ mice failed to raise their energy expenditure (EE) to the extent observed in their littermates; the increase of EE after CL-316,243 was 30.7% ± 8.2% compared with 64.3% ± 8.8% in the wild-type mice and 68.4% ± 4.4% in the *Mmut^+/–^* Tg^INS-Alb-Mmut^ littermates (*P* = 0.0018, 1-way ANOVA, Figure 3B), demonstrating decreased *β_3_*-AR–induced thermogenesis and suggesting impaired nonshivering thermogenesis in response to sympathetic nervous system stimulation.

In addition to the pharmacological adrenergic stimulus, we explored the response to a physiological cold stress. We exposed 16 male mice [4 wild-type (+/+); 7 heterozygotes, 3 +/–(–) and 4 +/–(+) — *Mmut^+/–^* Tg^INS-Alb-Mmut^; and 6 mutants — *Mmut^–/–^* Tg^INS-Alb-Mmut^] and 16 females [4 wild-type; 7 heterozygotes, 2 +/–(–) and 5 +/–(+); and 5 mutants] to 4°C (in a facility cold room) for 3 hours or until core body temperature reached 30°C. Rectal temperatures were monitored closely. In contrast with heterozygous and wild-type littermates, which were able to keep body temperature around 34.2–34.9°C, *Mmut^–/–^* Tg^INS-Alb-Mmut^ mice displayed striking sensitivity to the cold temperature, dropping to 33.9°C ± 1.5°C for males and 32.7°C ± 1.8°C for females at the 2-hour time point (body temperature and serum glucose changes were more prominent in the female animals, Supplemental Figure 2, F–H). Of note, 1 of the smaller female mice developed hypothermia (core temperature 29.6°C) and hypoglycemia (blood glucose 34 mg/dL) and was euthanized after 2 hours.

Targeted BAT gene expression in response to adrenergic stimulation after cold exposure was studied by quantitative real-time PCR (RT-PCR) at baseline (24°C) and after cold exposure (4°C) in sets of animals of comparable age and sex. Cold exposure triggers an increase in cAMP, which activates PPARγ coactivator 1-α (*Pgc1α*, also known as *PpargC-1α*), which, in turn, upregulates the transcription of uncoupling protein 1 (*Ucp1*) and type 2-iodothyronine deiodinase (*Dio2*). Ucp1 facilitates the uncoupling or “leaking” of protons produced by oxidative phosphorylation, leading to heat production instead of ATP synthesis (42), while Dio2 increases the activation of thyroxine to 3,5,3′-triiodothyronine, promoting mitochondrial thermogenesis in BAT (43). BAT from mutant mice maintained at room temperature showed significantly decreased expression of both *Ucp1* (69.8% ± 14.9% compared with wild-type controls, *P* = 0.0391, 1-way ANOVA, with Bonferroni correction for multiple comparisons) and *Dio2* (20.4% ± 11.3% compared with wild-type controls, *P* = 0.024) but not *Pgc-1α* compared to heterozygous and wild-type animals (Figure 3C). Another set of mice was exposed to 4°C for 3 hours. Quantitative RT-PCR showed significant upregulation of *Pgc-1α* in BAT of all animals exposed to cold. Both heterozygous and mutant mice showed lower responses compared with wild-type animals, but mutant mice exhibited similar induction of *Pgc-1α* as their heterozygous littermates. The most notable differences were observed in the transcriptional induction of *Ucp1* and *Dio2*. The stimulation of *Ucp1* in mutant mice was reduced to approximately 66% of the WT level (*P* = 0.0002, 1-way ANOVA, with Bonferroni correction for multiple comparisons), while the induction of *Dio2* was reduced by nearly 87% compared with WT controls (*P* < 0.0001) (Figure 3C). Lack of upregulation of *Ucp1* and *Dio2* may explain the insufficient mitochondrial “nonshivering” thermogenesis in BAT of mutant animals, resulting in inability to maintain body temperature after cold exposure. Significant upregulation at baseline and lack of further stimulation upon cold exposure was also observed for Ucp1 and other mitochondrial markers at the protein level (Supplemental Figure 5C).

To study the in vivo metabolic activity of BAT, we employed ^18^F-fluoro-d-glucose PET (^18^F-FDG-PET) studies in mice on HP diet that showed the abnormal BAT resembling WAT, followed by tissue collection and measurement of radioisotope biodistribution in different organs (Figure 3, D and E). A significantly decreased uptake of ^18^F-FDG in interscapular BAT was observed in mutant *Mmut^–/–^* Tg^INS-Alb-Mmut^ mice as opposed to their heterozygous littermates (Figure 3, D and E, *P* < 0.001), further verifying a dysfunctional brown fat. Surprisingly, increased ^18^F-FDG uptake was observed in the cardiac muscle in mutant animals as opposed to their heterozygous littermates (Figure 3, D and E, *P* < 0.001).

### Treatment effects of bezafibrates in MMA murine model.

To target extrahepatic PPARs, including PPARγ in adipose tissues, we chose bezafibrate, a pan-PPAR agonist, to stimulate adipose tissue mitochondrial biogenesis and potentially reverse the attenuated BAT function of *Mmut^–/–^* Tg^INS-Alb-Mmut^ mice. We used bezafibrate at 0.5% (corresponding to 300–500 mg/kg/d), which is the highest dose previously shown to be tolerable and efficacious in mice (44, 45).

**Figure 4:**
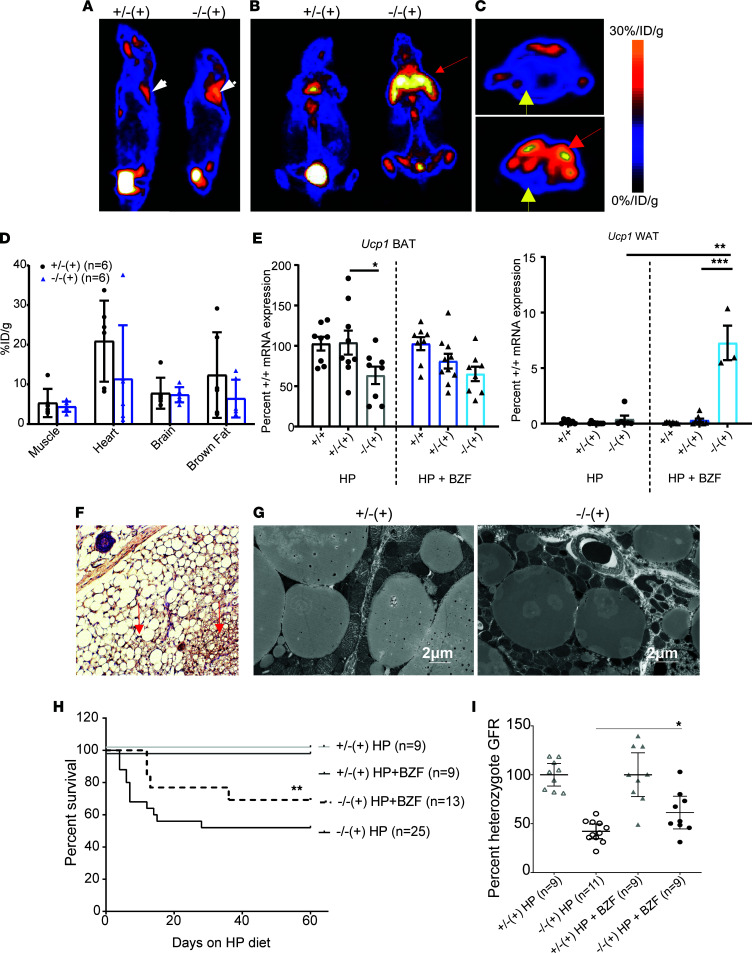
Bezafibrates reverse the brown fat mitochondrial dysfunction of mutant mice and induce beiging of subcutaneous fat depots in the regions of fat accumulation in participants with MMA. (**A**) Representative sagittal, (**B**) coronal, and (**C**) transaxial micro-PET images of mice after injection with ^18^F-FDG. Mice were fed high-protein chow with 0.5% bezafibrate (BZF) for 2 months. White arrowheads point to the interscapular brown fat, while the red arrow in the coronal section (**B**) points to a markedly enhanced uptake of radioactive label in the shoulder region of the mutant animals. This area appears superimposed to the normal location of BAT ^18^FDG uptake (red arrow in **C**). Yellow arrows (**C**) point to the myocardium of a heterozygote and mutant animal, respectively. (**D**) ^18^FDG biodistribution in various tissues shows no significant differences between control and mutant mice treated with BZF. (**E**) Quantitative RT-PCR of *Ucp1* mRNA expression in BAT (interscapular fat) and WAT (inguinal fat) showed no difference between mutant and control animals in BAT but a significant increase of Ucp1 expression in the subcutaneous WAT of the inguinal region in the mice fed high protein and 0.5% BZF (*P* < 0.0001 compared with heterozygotes, 1-way ANOVA with Tukey’s correction for multiple comparisons). (**F**) Immunohistochemistry staining for Ucp1 in subcutaneous adipose tissue of the shoulder region in a mutant mouse fed high protein and 0.5% BZF. Red arrows show multilocular lipid droplets staining densely for Ucp1 in the subcutaneous WAT resembling BAT (characteristic properties of beige fat). Blue arrow points to a hair follicle. Original magnification, 20×. (**G**) Ultrastructural studies demonstrated multinodular lipid droplets and numerous mitochondria with normal cristae in BAT from both heterozygous and mutant animals (scale bars: 2 μm). (**H**) *Mmut*^–/–^ Tg^INS-Alb-Mmut^ fed high-protein chow with 0.5% BZF show improved survival compared with high-protein chow only (Mantel-Cox survival curve comparison, *P* = 0.009). (**I**) Glomerular filtration rate (GFR) was measured with the FITC-inulin plasma decay method. Mutant mice fed high-protein chow showed a significantly reduced GFR (42.14% ± 3.35% compared with heterozygous mice on the same diet). GFR was improved to 61.29% ± 7.24% with BZF treatment (*P* = 0.02, compared with mutant mice on high protein only). *P < 0.05, **P < 0.01, ***P < 0.001.

Treatment with bezafibrate (0.5% in HP diet) for 2 months resulted in the restoration of radiolabeled glucose ^18^F-FDG uptake (PET) in the interscapular brown fat depot as well as normalization of uptake in the heart muscle in the mutant animals (Figure 4, A–D). In addition, there was significantly increased subcutaneous tissue glucose uptake in the shoulder/upper back area only in the mutant *Mmut^–/–^* Tg^INS-Alb-Mmut^ mice, resembling the distribution of subcutaneous fat expansion observed in patients with MMA (Figures 1 and 2). These observations were paralleled with a BAT *Ucp1* mRNA expression in the mutant mice that was not significantly lower than wild-type and heterozygous animals after bezafibrate therapy, as well as a significantly increased *Ucp1* expression in the subcutaneous inguinal adipose depot (*P* < 0.001, both compared with the mutant mice on HP as well as the heterozygotes on HP+bezafibrate, Figure 4E). Moreover, the subcutaneous adipose tissue in the shoulder region of the *Mmut^–/–^* Tg^INS-Alb-Mmut^ mice stained strongly positively for Ucp1, verifying the induction of beige or brown-in-white/brite adipose tissue in the area of high ^18^F-FDG uptake in the PET studies (Figure 4F). Last, bezafibrate also restored BAT ultrastructure. Mutant mice showed improved BAT mitochondrial numbers and structure, appearing almost indistinguishable from their heterozygous littermates (Figure 4G).

Although bezafibrate treatment was expected to improve the BAT phenotype, we were concerned about the systemic effects of inducing mitochondrial biogenesis/function in a disease model where mitochondrial reserve could be limited and added renal toxicity could be detrimental. It was therefore encouraging to observe an overall improvement in the health status and survival in *Mmut^–/–^* Tg^INS-Alb-Mmut^ mice exposed to the stress of HP diet with the addition of fibrate therapy. Bezafibrate treatment improved survival (*P* = 0.009) (Figure 4H) and attenuated the decline in the glomerular filtration rate (GFR) typically observed in *Mmut^–/–^* Tg^INS-Alb-Mmut^ mice 2 months after HP exposure (*P* = 0.019) (Figure 4I). Furthermore, in support of decreased renal injury, a decline in percentage wild-type lipocalin-2 mRNA expression was observed in the kidneys of bezafibrate-treated mutant animals, along with decreased serum methylmalonic acid concentrations and normal proximal tubule mitochondrial ultrastructure (Supplemental Figure 3, A–D). Interestingly, reducing the stress of cold exposure by housing on heating pads alone had a similar effect on renal disease and serum methylmalonic acid concentrations to treatment with bezafibrate or the previously reported treatment with antioxidants (18), suggesting a strong effect of minimizing stress on mouse metabolic health. Plasma creatinine was increased in bezafibrate-treated animals (Supplemental Figure 3C), which could reflect improved FFM/lean mass accretion, given the observed weight gain.

### FGF21 and acyl-CoA accretion are associated with beiging of subcutaneous adipose depots in MMA.

Dysregulation of FGF21 was previously described in MMA murine models and patient cohorts and shown to correlate with disease severity and multiorgan complications (15, 27). FGF21 is a downstream target of PPARα and sirtuin-1 (SIRT1), and has pleiotropic metabolic effects, including antihyperglycemic, antihyperlipidemic, and thermogenic properties (46–49). We therefore sought to further explore correlations of FGF21 with the lipodystrophy phenotype and other adipokines in our cohort.

**Figure 5:**
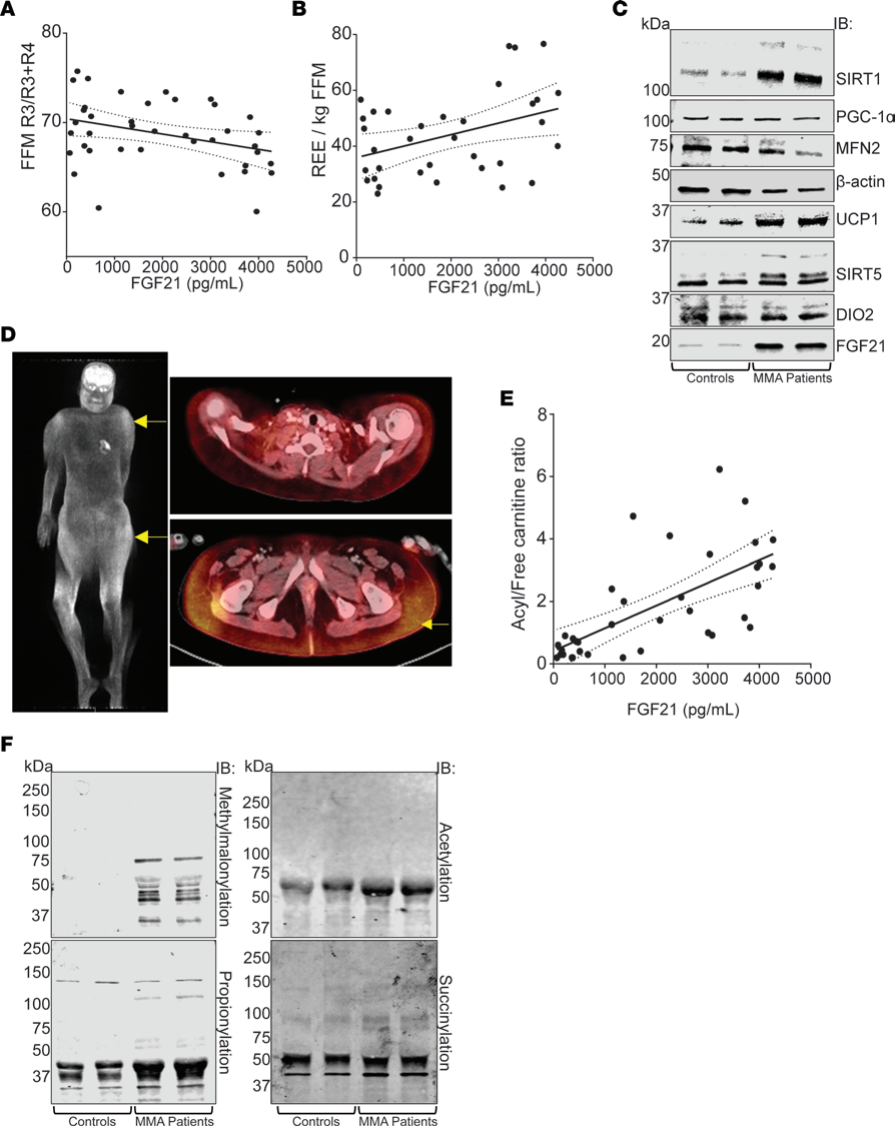
FGF21 is associated with beiging of subcutaneous fat depots and acyl-CoA trapping. (**A**) In the MMA patient cohort, FGF21 concentrations correlated with the abnormal body composition observed in lower extremities and (**B**) with an increased energy expenditure per kilogram fat-free mass. (**C**) Immunoblotting of selected mitochondrial or beige fat markers is depicted in subcutaneous adipose tissues of 2 young adult female control participants undergoing postmortem examinations at the NIH Clinical Center, and 2 female participants with *mut^0^* MMA: upper anterior chest wall subcutaneous fat of the index case obtained during a central line removal and the upper arm subcutaneous fat of a woman in Figure 1D, obtained during a postmortem exam at age 34 years. While no significant differences were observed in the expression of PGC-1α, MFN2, and DIO2, higher density bands were evident in patients with *mut^0^* MMA for SIRT1, UCP1, SIRT5, and FGF21. (**D**) Combined PET/CT imaging study showing extensive subcutaneous uptake of ^18^F-FDG (yellow arrows), over the shoulders and gluteal area/upper thighs in a 13-year-old girl with *mut^0^* MMA. Standard Uptake Values of ^18^F-FDG are provided in Supplemental Figure 4. (**E**) Circulating FGF21 in the MMA patient cohort correlated with the acyl-CoA species accumulation (propionyl-C3 and methylmalonyl-C4DC carnitine) reflected in the increased circulating acyl- to free carnitine plasma ratio. (**F**) Posttranslational modifications (PTMs), including methylmalonylation, propionylation, acetylation, and succinylation, are shown in subcutaneous tissues of participants with *mut^0^* MMA compared with controls. The biggest differences were observed in lysine residue methylmalonyl — PTMs that were completely absent from control WAT.

Circulating FGF21 in patients with MMA correlated negatively with FFM R3/R3+R4 (*r* = –0.345, *P* = 0.0311, *R*^2^ = 0.119), suggesting a correlation with subcutaneous adipose accumulation in the proximal lower limbs (Figure 5A). Moreover, FGF21 correlated positively with the resting EE per kilogram FFM (*r* = 0.395, *P* = 0.0205, *R*^2^ = 0.1156) (Figure 5B). FGF21 induction as well as induction of other mitochondrial markers, including UCP1, SIRT1, and SIRT5, was further verified by Western immunoblotting using subcutaneous adipose tissue of patients with MMA (Figure 5C), indicating the presence of white fat with brite or beige fat properties (50, 51). Last, “beiging” of subcutaneous adipose tissue in MMA was characterized by the markedly increased ^18^F-FDG uptake in subcutaneous adipose depots of a 13-year-old *mut^0^* MMA Hispanic girl (compound heterozygous variants in *MMUT*: c.1106G>A; p.Arg369His and c.1196_1197del; p.Val399GlufsTer24), who underwent a radiolabeled glucose (^18^F-FDG) PET study to rule out an ectopic parathyroid hormone–secreting tumor suspected because of hypercalcemia (Figure 5D and Supplemental Figure 4). The distribution of increased ^18^F-FDG uptake in this patient showed a striking similarity to *Mmut^–/–^* Tg^INS-Alb-Mmut^ mouse PET studies (Figure 4, A–C).

Moreover, a strong correlation was observed between FGF21 and the total acylcarnitine/free carnitine ratio (*r* = 0.675, *R*^2^ = 0.455, *P* < 0.0001, Figure 5E), which made us hypothesize that PTMs may be involved with FGF21 or Pgc-1α transcriptional cascade dysregulation in *Mmut^–/–^* Tg^INS-Alb-Mmut^ mice. Increased PTMs, especially methylmalonylation, were indeed observed in both human and mouse adipose tissues (Figure 5F and Supplemental Figure 5, B and D). Cold exposure caused significant changes in the PTM landscape evident in WAT extracts from *Mmut^–/–^* Tg^INS-Alb-Mmut^ mice (Supplemental Figure 5B).

Given the reported antidiabetogenic and antiinflammatory effects of FGF21, we explored how other adipokines/cytokines might be affected by the extreme and chronic elevations of FGF21 in the MMA patient cohort and whether adipose accumulation was associated with inflammatory markers/cytokines causally linked to insulin resistance, metabolic syndrome, and T2DM. We matched a smaller group of 20 patients with both *mut^0^* (low to no methylmalonyl-CoA enzymatic activity) and *mut^–^* (moderate methylmalonyl-CoA activity) MMA, 10 with very high FGF21 and 10 with lower values, with a subset of 20 sex-, age-, and BMI-matched control individuals recruited from an obesity study and performed a screen using the SomaScan platform, as previously described (52–54). Significantly up- and downregulated serum protein biomarkers are depicted in a volcano plot (Supplemental Figure 6B). Patients with MMA, despite massive elevations in plasma mitochondrial markers, FGF21, and GDF15 concentrations (Supplemental Figure 6A), had overall lower inflammatory cytokine levels, including adipokines typically elevated in obesity and the metabolic syndrome (IFN-γ, TNF, IL-6, IL-12, IL-1B) (55, 56) compared with the age-, sex-, and BMI-matched control subset (Supplemental Figure 6, C and D, and Supplemental Table 2). Independent validation of several biomarkers using specific ELISAs, including lipocalin-2 (NGAL), GDF15, and pro-BNP, showed a strong correlation with the SomaScan values (data not presented).

## Discussion

In the current work, we combine observations in patients and disease-specific murine models to characterize what we believe to be a novel obesity endophenotype of MMA. Adipose tissue is second only to skeletal muscle in its capacity to catabolize BCAAs (57–59), and MMA provides a unique opportunity to explore the impact of defective valine/isoleucine oxidation on associated physiology (4, 9, 10, 60). Previous studies showed a downregulation of BCAA degradation pathway genes, including *PCCA/B* and *MMUT*, in insulin resistance/T2DM (9), while a GWAS noted an association between insulin resistance and a region downstream of *MMUT* (61). Furthermore, impaired glucose homeostasis, with disturbed lipid oxidation and triglyceride accumulation in skeletal muscle of *Mmut*^+/–^ mice compared with wild-type littermates, was observed after high-fat feeding (9). However, although obesity and increased body fat mass have been recognized in MMA (21, 22), large patient registries and natural history cohorts do not report insulin resistance and metabolic syndrome/T2DM among the chronic complications of the disease (15, 26, 62, 63). A smaller study employing oral glucose tolerance testing further supports a benign metabolic phenotype in patients with *MMUT* MMA and a different obesity pattern compared with patients with propionic acidemia (14).

We show that young children and adults with the most severe enzymatic subtypes, *mut^0^* and *cblB* MMA, can develop a distinct lipodystrophy characterized by the expansion of subcutaneous adipose tissue in the upper body (back, arms, upper legs), along with loss of fat in the lower extremities and scarcity of visceral fat. Their subcutaneous adipose tissue displays properties of beige (or brite) adipose tissue, including the expression of markers typically found in brown fat (UCP1, FGF21, SIRT1 and -5) and increased ^18^F-FDG uptake on PET imaging. This acquired partial lipodystrophy closely resembles the phenotype of multiple symmetric lipomatosis (MSL; OMIM #151800; Madelung’s disease; Brodie, Launois-Bensaude, or Ekbom’s syndrome) (64–67), which was originally described in men of Mediterranean origin with chronic alcoholism (68–70). An MSL-like phenotype has also been observed in a subset of patients with highly active antiretroviral therapy–associated lipodystrophy syndrome (71, 72), in individuals with myoclonic epilepsy with ragged-red fibers (73, 74), and patients with biallelic variants in mitofusin-2, typically including a p.Arg707Trp allele (*MFN2*) (75, 76). Some studies on MSL suggest a benign metabolic phenotype (77), with increased circulating adiponectin levels, increased resting energy expenditure, expression of markers of brown fat (typically UCP1) and increased ^18^F-FDG uptake in the expanded subcutaneous fat depots, and in the case of *MFN2*-MSL, increased circulating FGF21. Collectively, the patient observations suggest that a metabolically active/beige, rather than white, adipose tissue is proliferating in the lipomas (34, 67, 76). Despite the similarities, there are some notable differences between the lipodystrophy of patients with MMA and MSL, including the older age of onset in MSL with very rare reports in pediatric patients (78, 79), the frequent mediastinal and neck expansion of adipose tissue requiring in some cases tracheostomy, and the associated sensorimotor axonal neuropathy with secondary foot contractures. Furthermore, MSL is associated with increased resting EE per kilogram of skeletal muscle mass (34) whereas patients with MMA have decreased resting EE (21), though a trend to higher resting EE/kg FFM was observed in patients with the higher FGF21 concentrations (Figure 5B). Furthermore, patients with MMA appear to exhibit higher circulating FGF21 concentrations (Table 1 and Supplemental Figure 6) compared with *MFN2*-associated MSL, where the mean was reported as 385 ± 181 pg/mL (mean ± SD) (76).

We used a Tg MMA murine model to further explore the physiology of MMA lipodystrophy and study the pathology, ultrastructure, and transcriptional response to cold or HP stress exposure of BAT and WAT. We showed that abnormal BAT pathology and function (response to β_3_-adrenergic stimulation, ^18^F-FDG uptake on PET imaging, and transcriptional activation of mitochondrial thermogenesis to environmental cold exposure) result in deficient nonshivering thermogenesis. Due to their reduced tolerance of the slightly colder room temperature, mice preserve energy by decreasing their activity levels, similar to the hypoactivity observed in a *Mmut*-ko/ki murine model (80). Interestingly, the *Mmut*-ko/ki murine model also showed signs of whitening of the BAT, cold intolerance (81), and decreased cardiac systolic function after HP diet exposure. Although we have not performed echocardiograms in the current study, we observed a marked ^18^F-FDG uptake in the heart muscle of the *Mmut*^–/–^ Tg^INS-Alb-Mmut^ mice in our PET imaging studies. Whether this is a result of a higher heart rate in the mutant animals or a switch to glycolysis because of dysfunctional mitochondrial fatty acid oxidation in the MMA myocardium remains an intriguing question for future studies. Last, *Mmut*^–/–^ Tg^INS-Alb-Mmut^ mice display a low total EE/kg of FFM similar to what we previously described in our MMA patient cohort (21), closely replicating these aspects of the disease phenotype.

A compensatory activation of beiging in WAT in response to BAT paucity/dysfunction has been previously observed in genetically engineered models (82, 83), similar to the *Mmut*^–/–^ Tg^INS-Alb-Mmut^ mice and patients, who show increased expression of Ucp1 and other mitochondrial markers in WAT (Supplemental Figure 5A and Figure 5C). PPAR agonists can induce browning of WAT, and administration of a PPARα agonist (fenofibrate) showed effective control of hypertriglyceridemia and reduction of adipose tissue in a patient with MSL (66). Bezafibrates are pan-PPAR agonists (α, β/δ, and γ), targeting both PPARα in the liver, as well as PPARγ in adipose tissue, and were shown to be superior in stimulating both FGF21 and PGC-1α to induce mitochondrial biogenesis and therefore would be better aimed at reversing the abnormal brown fat phenotype observed in the *Mmut*^–/–^ Tg^INS-Alb-Mmut^ mice (84–86). Bezafibrates were shown to restore levels of *PGC-1α*, PPARs and downstream genes (*SIRT1*, *UCP1*) with reversal of a similar BAT phenotype to the *Mmut*^–/–^ Tg^INS-Alb-Mmut^ mice in mouse models of neurodegenerative diseases (87) and to induce hepatic autophagy and reduce lipogenesis and inflammation in GSD-1 murine and canine models (88). Moreover, they can ameliorate disease phenotype, including cardiac pathology, in various mitochondrial disease and RASopathy murine models (89–91). However, there have been mixed results from clinical trials of selected fatty acid oxidation disorders and mitochondrial myopathies and toxicity concerns raised, which are species specific (92–97). Bezafibrate treatment in the *Mmut*^–/–^ Tg^INS-Alb-Mmut^ mice improved survival, restored the mitochondrial ultrastructure and metabolic activity of BAT (improved ^18^F-FDG uptake in the PET study), and increased Ucp1 expression in the WAT of treated animals, associated with an increased ^18^F-FDG uptake in subcutaneous fat tissue in a distribution pattern similar to the expanded subcutaneous fat depots seen in patients. Importantly, treated mice showed an attenuation of the decline in GFR measurements induced by HP diet, which was encouraging, given the known renal side effects of this class of medicines. Although longer experiments will be necessary to establish efficacy and safety, our findings suggest that induction of mitochondrial biogenesis targeting adipose tissue with selective or newer PPARδ agonists (98) could offer therapeutic benefit to patients with MMA.

The elevated circulating concentrations of FGF21 in MMA participants and the correlation with the plasma acyl/free carnitine ratio (15, 99), along with the high expression of FGF21 and other mitochondrial markers (SIRT1, -5, UCP1) and increased PTMs in adipose tissues from patients and *Mmut*^–/–^ Tg^INS-Alb-Mmut^ mice, support that the acyl-CoA accretion (propionyl and methylmalonylation) observed in MMA plays a key role in disease pathophysiology (20). Further investigations are needed into the role of PTMs as a putative common factor between MMA, chronic alcoholism, and MSL and their impact on FGF21 and MFN2 regulation. It is intriguing that similar PTMs to MMA adipose tissue and liver, including increased acetyl/propionylation and decreased succinylation, have been observed in livers from mice exposed to alcohol (100), and urinary excretion of propionate and methylmalonate was demonstrated in acute or chronic alcohol exposure in rat studies (101). However, no PTM studies have been performed in adipose tissue of lipomatosis subjects linked to chronic alcoholism or MSL as of yet. While we did not observe abnormal expression of MFN2 in our patient adipose tissues, increased MFN2 expression compared with heterozygous littermates was observed in mouse WAT and BAT. There was no transcriptional dysregulation of *Mfn2* or other lipodystrophy genes in previously published liver or kidney tissue expression studies (15, 18) or in MMA patient-derived kidney cells (19). However, it is possible that low abundance and PTM of SIRT5 and or SIRT1, previously demonstrated in MMA mouse models (20), as well as other vitamin B_12_ metabolism defects (102), are linked to abnormal *MFN2* function (103, 104).

This study illustrates the critical role of the vitamin B_12_–dependent terminal steps of valine and isoleucine oxidative pathways in the mitochondrial physiology of WAT and BAT. It adds BAT to the list of organs manifesting cell-autonomous mitochondrial dysfunction in MMA and shows that induction of key metabolic regulator FGF21 is associated with markers of CoA accretion and an expansion and beiging of subcutaneous fat depots. Notably, the subcutaneous fat accumulation in MMA is not associated with abdominal-visceral obesity or an inflammatory cytokine profile that typically conveys a high risk for insulin resistance and the metabolic syndrome/T2DM. Improving mitochondrial biogenesis and reducing acyl-CoA accretion by small molecules in affected organs, including the adipose tissue, could provide therapeutic benefit in MMA and perhaps other forms of obesity associated with inborn errors of metabolism.

## Methods

### Clinical studies.

MMA patient studies were approved by the NIH Institutional Review Board as part of the natural history protocol “Clinical and Basic Investigations of Methylmalonic Acidemia and Related Disorders” (ClinicalTrials.gov identifier: NCT00078078) and performed in compliance with the Helsinki Declaration. Patients or their parents/legal guardians provided informed consent. The specific subtype (*mut^0^*, *mut^–^*, *cblA*, *cblB*) of isolated MMA was assigned based on molecular genetic analysis of the genes *MMUT*, *MMAA* and -*B*, and, when available, cellular biochemical studies in fibroblasts (David Rosenblatt, McGill University, Montréal, Québec, Canada), as previously described (105, 106). Study participants were evaluated at the NIH Clinical Research Center, when clinically stable. We analyzed DXA (Hologic Delphi A; Hologic) scans from 46 patients with isolated MMA (32 *mut/MMUT* (complementation subtype/gene), 8 *cblA/MMAA*, and 6 *cblB/MMAB*), where whole-body composition in grams of FM and fat-free (or lean) body mass (FFM) were quantified. Moreover, 4 subregions were analyzed for each patient (upper and lower arm [R1 and R2] and leg [R3 and R4] segments respectively, based on bony landmarks) and ratios of upper to whole limb fat were calculated (R1/[R1+R2] for upper and R3/[R3+R4] for lower limb). Two independent observers drew the R1–R4 regions separately for 20 patients with MMA and 25 controls to assess interobserver variability, and the average numbers were included in the final analyses. Coefficients of variation were very low (for fat mass [g] in patients with MMA: R1: 3.09% ± 2.45%, R2: 2.64% ± 1.63%, R3: 3.21% ± 2.43% and R4: 1.53% ± 3.10%; in the control individuals, R1: 2.52% ± 1.71%, R2: 3.19% ± 2.70%, R3: 3.45% ± 2.17% and R4: 0.96% ± 1.63%). Clinical data derived from the natural history protocol were available for correlations, including dietary information (kcal/d, complete/deficient protein g/kg/d, and individual amino acid intake; ref. 22), resting EE measured by open-circuit indirect calorimetry (21) (Deltatrac equipment; VIASYS), plasma quantitative amino acids, acylcarnitine profiles, methylmalonic acid, adiponectin, leptin, renal function markers, and other biochemical data. Moreover, SomaScan v1.3 aptamer-based multiplex proteomic platform of 1,129 targets was employed (SomaLogic), at the Trans-NIH Center for Human Immunology, to investigate cyto/adipokines or other immune/inflammation biomarkers associated with the extreme elevations of methylmalonic acid, acylcarnitine species, and FGF21 described in MMA mice and patients with MMA (15, 27).

MMA patient data were compared with 99 generally healthy controls with similar age, sex, race and ethnicity, and BMI, obtained with the same DXA Hologic Delphi system. We aimed for an approximately 2 controls/1 patient match from patients enrolled in NIH protocols studying children and young adults who may have obesity (ClinicalTrials.gov IDs: NCT00001522, NCT00005669, NCT00030238, NCT00320177, NCT00263536, and NCT00315172). Demographic and clinical information for the MMA cohort and controls is provided in Table 1.

Research fat biopsies and ^18^F-FDG-PET/CT studies were not part of the scheduled research protocols. Biopsy samples were obtained during surgery for central catheter removal (index case) or from research autopsies. The PET/CT imaging data included here were obtained from outside institutions and performed as a result of clinically indicated testing and subsequently shared with the study team after obtaining consent.

### Mouse studies.

Animals were housed in an Association for Assessment and Accreditation of Laboratory Animals–accredited specific pathogen–free facility. Generation and characterization of the liver- (*Mmut*^–/–^ Tg^INS-Alb-Mmut^) and muscle- (*Mmut*^–/–^ Tg^INS-MCK-Mmut^) Tg mouse models have been described previously (18). *Mmut*^–/–^ Tg^INS-Alb-Mmut^ were used for the cold stress experiments as well as for the HP chow with and without bezafibrate treatment experiments in the current work. Methods describing mouse physiology using metabolic chambers, ^18^F-FDG-PET imaging, transcriptional response to cold stress, Western blotting, histology, immunohistochemistry, and electron microscopy are provided in the Supplemental Methods section.

### Statistics.

All data were prepared for analysis with standard spreadsheet software (Microsoft Excel). Statistical analysis was done with Microsoft Excel, Prism 4.0 (GraphPad), or the IBM SPSS statistics software, version 21. Data are presented as the mean ± SEM or SD, as indicated. Statistical comparisons were performed using unpaired 2-tailed *t* test or 1-way ANOVA followed by Bonferroni or Tukey-Kramer post hoc test for multiple comparisons. For nonparametric comparisons, Kruskal-Wallis with Dunn’s correction were employed. When data were not normally distributed or groups were of unequal size, the Kruskal-Wallis 1-way ANOVA testing was performed. Pearson’s correlation coefficient and linear regression were employed for correlations. A *P* value of less than 0.05 was considered significant.

### Study approval.

Patients with MMA were enrolled via a longitudinal natural history protocol (ClinicalTrials.gov ID: NCT00078078); controls with obesity were enrolled via other protocols that obtained DXA scans (ClinicalTrials.gov ID: NCT00001522, NCT00005669, NCT00030238, NCT00320177, NCT00263536, and NCT00315172). All were approved by the NIH Institutional Review Board (FWA 00005897). Written informed consent was obtained from all participants or their legal guardians. Assent forms were signed by child participants deemed cognitively able to do so. Written informed consent was received for the use of photographs.

Animal studies were performed in agreement with NIH guidelines and with the approval of the Animal Care and Use Committee of the National Human Genome Research Institute (NHGRI) and National Institute of Diabetes and Digestive and Kidney Diseases (NIDDK).

### Data availability.

Data are available in the Supporting Data Values XLS file; deidentified human participant data, animals, reagents, or protocols are in Supplemental Methods or can be made available from the corresponding author upon request.

## Author contributions

IM designed and supervised the study; provided clinical care; performed murine model experiments, including cold-challenge studies; performed bezafibrate treatment, PET imaging, mouse and human histology, proteomic screening, figure preparation, and statistical analyses; and wrote the paper. JRS performed murine model experiments, including cold-challenge studies; performed bezafibrate treatment, PET imaging, mouse and human histology, gene expression studies, and data analysis; and edited the paper. PSEH performed protein expression and PTM studies in murine and human tissues, performed figure preparation, and edited the paper. MWE, CW, YPK, ARP, and SAK performed various gene and protein expression experiments, human sample immunoassays, immunohistochemistry, DXA data analysis, and figure preparation and edited the paper. OG performed metabolic cage physiology studies, body composition measurements, data processing, and analysis in mice in a dedicated mouse metabolism core of NIDDK. MKC, SM, JCR, and JAY shared obese matched control clinical data, supervised DXA subregion analysis, and shared samples for adipokine and proteomics screening. XSC supervised the mouse PET imaging and radioisotope biodistribution studies. JLS coordinated the clinical study, obtained informed consent for participants, provided genetic counseling, analyzed clinical data, and edited the paper. PMZ, VH, and MGT performed mouse and human histology and electron microscopy studies and helped with interpretation and figure preparation. LAF and KYC helped with human ^18^F-FDG-PET image analysis and figure preparation. HJV and AH referred patients and shared serum samples for biomarker validation. CAS and RJB provided clinical care and endocrine consultation for the index patient. SDV and GTB shared human ^18^F-FDG-PET and referred patients to the study. CPV designed and supervised clinical and mouse research studies and wrote the paper.

## Supplementary Material

Supplemental data

Unedited blot and gel images

Supporting data values

## Figures and Tables

**Table 1:**
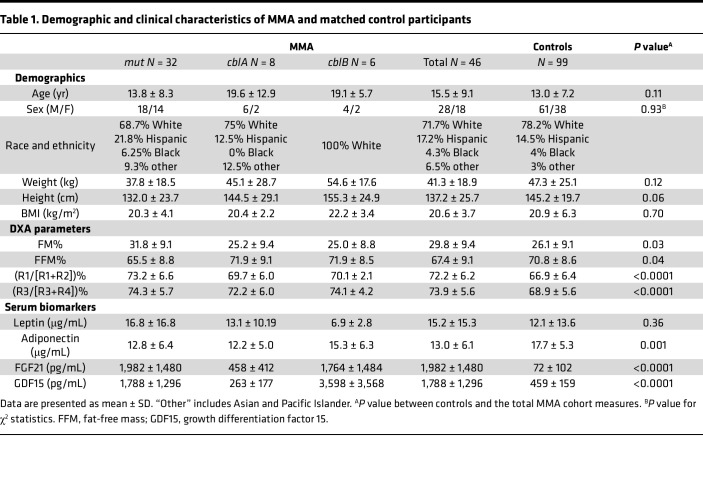
Demographic and clinical characteristics of MMA and matched control participants

## Version 1 01/25/2024

In-Press Preview

## Version 2 02/22/2024

Electronic publication
