# Supplementary material for: Lipodystrophy in methylmalonic acidemia associated with elevated FGF21 and abnormal methylmalonylation
Source: JCI Insight. 2024 Feb 22;9(4):e174097. doi: 10.1172/jci.insight.174097 (PMC10967474; doi:10.1172/jci.insight.174097)
Supplement: Supplemental data [file jciinsight-9-174097-s146.pdf]

SUPPLEMENTAL INFORMATION:

PATIENT STUDIES:

Index case clinical details and laboratory evaluation: The index patient was diagnosed prenatally due to a similarly affected older sister, who was compound heterozygote for the following pathogenic variants in *MMUT*: c.323G>A, p.Arg108His and and c.1867G>C, p.Gly623Arg diagnosed as mut^0 based on ^{14}C-propionate incorporation studies and B12 responsiveness in fibroblasts (case WG2515)(1). Except for the similarly affected sister, and father's history of T2DM, there was no other family history of note and no known consanguinity. Pre-emptive management was successful in preventing a severe neonatal hyperammonemic coma and she had a relatively benign course until age 3-5y when she suffered recurrent metabolic decompensations during intercurrent illnesses, which is typical for mut^0 MMA patients. She required a gastrostomy and central line access and suffered several central line-sepsis episodes in addition to classic MMA complications, such as recurrent pancreatitis, chronic kidney disease and a metabolic stroke in her basal ganglia (globus pallidi). At age 6 years, her weight was 29.2kg (weight-for-age *Z*-score: 2.06), height 110.2cm (height-for-age *Z*-score: -0.89), and BMI was 24.58kg/m2 (Z-score: 2.62). She was Tanner stage I, and there were no violaceous striae, excessive bruising, or hypertension. She had a movement disorder consistent with the history of basal ganglia metabolic stroke. Her medications included: Epoetin alfa 2,000 units and G-CSF 125 μg, Bicitra, Polycitra K, Calcium glubionate, Levocarnitine 2.5gr, Coenzyme Q10 30 mg, Zantac 150 mg, Nitrofurantoin 25 mg. Her diet consisted primarily of elemental amino acid formula (Neocate, Nutricia North America, Gaithersburgh, MD) 210 g mixed in 2200 cc of normal saline with no added propiogenic amino acid deficient medical foods. Her total diet provided 1400kcal/d and 0.95g/kg/d of complete protein from formula and oral intake.

Serum MMA was 984μmol/L (normal <0.4), plasma lactate, processed accurately and repeated twice, was 7.8 mmol/L (normal range 0.5-2.2), pyruvate 4.1mg/dL (normal range 0.7-1.4), liver function tests were normal. Non-fasting serum insulin level was 6.8 U/mL (normal range 6-27) with a glucose of 160mg/dL. Hemoglobin A1C was less than 4%. Leptin was 20.4ng/mL, adiponectin was 30.5ng/mL. TSH was 3.69μIU/mL

(normal 0.4-4.0), T3 was 76ng/dL (90-215), thyroid binding globulin was 7μg/mL (13-39), IgF1 was 68ng/mL. FSH was 3, LH <1U/L, free testosterone less than 0.1ng/dL, total testosterone less than 7.0ng/dL. Serum estradiol was less than 10pg/mL, DHEAS was less than 15μg/dl, DHEA was 70ng/dL, androstenedione was less than 15ng/dL. Diurnal cortisol included an 11:30 p.m. cortisol of 2.5, 12:00 a.m. of 3.1, 7:30 a.m. of 5.5 and 8:00a.m. of 11.7 μg/dL. Repeat diurnal cortisols include 11:30 p.m. value of 3.2, 12:00 a.m. value of 3.2, 7:30 a.m. value of 8.3, and 8:00 a.m. value of 8.4 μg/dL. ACTH was 14.5 pg/mL at 7:45 in the morning. Dexamethasone-suppressed CRH stimulation test was performed with ACTH values of 12.4, 15, 57.2, 54.8, and 53.8 pg/mL, and cortisol values of 9.1, 8.4, 13.2, 18 and 11.9nmol/L at -50, +15, +30, and +45 minutes respectively. Peak plasma cortisol < 38 nmol/L was normal (1.4 μg/dL) (100 nmol/L=3.6 μg/dL).

Ultrasound of the abdomen showed normal spleen, kidneys and liver, with increased subcutaneous fat. Ultrasound of the dorsocervical fat area showed increased vascularization (Supplemental Figure 1D). Whole body DEXA was performed with a BMD corrected for height Z-score of 1.3.

Subcutaneous tissue obtained during a port-a-cath removal showed mildly positive staining for uncoupling protein -1 expression (UCP1) and rare mitochondria with abnormal ultrastructure (Supplemental Figure 1E and F).

Patient ^{13}F-FDG-PET study: Approximately 60min after the intravenous administration of 5.788mCi F-18-fluoro-2-deoxyglucose, PET/CT was performed form the base of the skull to the thighs. The blood glucose was 97mg/dl. The patient had hemodialysis 5hrs prior to injection. Great care was taken to ensure that no glucose containing fluids were used during dialysis. The patient's gastrostomy tube feeds were also withheld. PET and POCT fingerstick glucose were performed in Nuclear Medicine, 300 Longwood Ave, Boston, MA.

Dual-Energy X-ray Absorptiometry (DXA) analysis: Whole body scans were performed using DXA (Hologic Delphi A; Hologic, Bedford, MA) to quantify the full-body mass distribution for fat, fat-free (lean) mass, bone and total. Subtotal mass represents full body minus the head measurements. Using the subregion analysis tool, customized regions of interest (ROIs) were drawn to capture 4 segments: right upper arm, right forearm, right thigh and right shank (Figure 2A and Supplemental Table 1). To assess the between-day intra-tester and

inter-tester reliability for the cross-section analysis of appendicular segments, all DXA scans subregions were drawn and analyzed twice by the same observer, while a subset of 50 scans (20 patient and 25 controls) were re-analyzed separately by two different observers. Coefficients of variation (CV) were calculated using IBM SPSS Statistics Version 21.0, Chicago, IL. Coefficients of variation below 5% were considered highly reliable. (Supplemental Table 1)

SomaScan assay: Large-scale proteomic analysis was performed using the SomaScan 1.3k Assay (SomaLogic, Boulder, CO, USA) at the Trans-NIH Center for Human Immunology, Autoimmunity, and Inflammation (CHI), as described previously(2). This aptamer-based assay can detect 1305 protein analytes in human serum, including cytokines, hormones, growth factors, receptors, kinases, proteases, protease inhibitors, and structural proteins. A complete list of analytes measured can be found at http://somalogic.com/wp-content/uploads/2017/06/SSM-045-Rev-2-SOMAscan-Assay-1.3k-Content.pdf. Concentrations are measured as relative fluorescence units. A subset of 20 MMA patient samples were used, 10 with high and 10 with low elevations in plasma FGF21 concentrations, previously measured by quantikine ELISA (R&D Systems, DF2100); control set included plasma samples from 20 age, gender, BMI matched controls. Specimens (50mL) were diluted to three concentrations (0.005%,1%, and 40%) to separate groups of high, medium, and low abundance proteins, respectively, and then combined with dilution-specific SOMAmers. Quality controls (QC) and calibrators provided by SomaLogic were run together with internal site QC samples. Data generated from these samples were used to assess interassay variability, as described previously. Analysis of the data was performed with Partek platform and Ingenuity Pathway Analysis (IPA). Data were analyzed using SomaSuite version 1.0.3 (NEC Corporation, Minato, Tokyo, Japan) and web-tools developed by CHI (https://foocheung.shinyapps.io/adat_v02/andhttps://foocheung.shinyapps.io/plotterII/)(3, 4). Unpaired t-tests for two group comparison false discovery rate (FDR) for correction for multiple comparisons were performed. Pearson correlation was performed using an online tool developed by CHI.

MOUSE STUDIES: Mice were maintained on a 12:12-h light-dark cycle, and all experiments were performed in agreement with National Institutes of Health guidelines and with the approval of the Animal Care and Use Committee of NHGRI and NIDDK.

Metabolic phenotyping. Indirect calorimetry was performed using an eight-chamber Oxymax system (Columbus Instruments, Columbus, OH) with one mouse/chamber and by testing transgenic *Mmut*$^{-/-}$;Tg$^{\text{INS-Alb-}\mathit{Mmut}}$ mice (n=5), simultaneously with littermate controls, *Mmut*$^{+/-}$;Tg$^{\text{INS-Alb-}\mathit{Mmut}}$ (n=12) and wild type littermates (n=6). Female mice, 2.8-3.1 months old, were used for these experiments. Experimental design was as follows: Day 1: mice were adapted to metabolic chambers at 24°C; day 2: data were collected for 24h at 24°C; day 3: data were obtained for 24h at 30°C; day 4: mice were injected intraperitoneally with the β_3-selective adrenergic agonist CL-316,243 (1mg/kg in saline) as described previously (5). Activity (total and ambulatory) was determined by infrared beam interruption (Opto-Varimex mini, Columbus Instruments, Columbus, OH). Mice had free access to regular chow (7022 NIH-07 diet, 29% kcal from protein, 56% from carbohydrates and 15% , Harlan Laboratories, Madison, WI) and water. Total O_2 consumption was calculated as the average of the points with fewer than six ambulating beam breaks per interval and omission of the first hour of the experiment. The respiratory exchange ratio (RER; the ratio of CO_2 produced to O_2 consumed) was calculated using the same data points. Oxidation of carbohydrate produces an RER of 1.00, whereas FA oxidation results in an RER of 0.70. Results for O_2 consumption were normalized to (body weight) ^ 0.75 . The effect of CL-316,243 on metabolic rate was measured with each mouse serving as its own control. Mice were placed into the calorimetry chambers (prewarmed to 30°C) at 9:00 AM and baseline data were collected. CL-316,243 was injected (1 mg/kg ip) 3h later, and after a 1h delay data were collected for 3h. Body composition was measured in non-anesthetized mice with time domain Echo MRI 3-in-1 analyzer (Echo Medical Systems, Houston, TX).

Cold exposure experiment: Core body temperature and blood glucose were monitored hourly or every 30min if the temperature was reduced, by a rectal temperature probe (RET3 probe connected to a Thermocouple thermometer #BAT-12R, World Precision Instruments, FL). Three hours after cold exposure mice were anaesthetized inside the cold room, by injection of sodium pentobarbital (30 mg/ml in 0.9% NaCl; 1.5 ml/kg of body mass, intraperitoneal) and, once locomotor activity had ceased, retroorbital bleed was obtained, and

brown and white fat pads were dissected and snap frozen for subsequent gene expression analysis. Mean temperature change (%change from baseline) was monitored at hourly intervals. Temperature drop was not significantly different between the different genotypes, although the trend was bigger for female mutant mice at the 3hrs time point. Another set of mice kept at room temperature (24 ± 1°C; controls) were euthanized for tissue collection as controls.

^{18}F-FDG-PET studies: FDG-PET studies were performed using an Inveon small-animal PET scanner (Siemens Preclinical Solutions). ^{18}F-FDG (18F-fluoro-D-glucose) was administered intraperitoneally to experimental mice with a dose of 11.1 MBq. Mice were kept fasting 3h prior to the PET studies. One hour after the tracer had been injected, a 15 min static PET scan was performed under isoflurane anesthesia with a heating pad to keep anesthetized animals at 23-25^{0}C. The images were reconstructed using a two-dimensional ordered-subset expectation maximum (2D OSEM) algorithm. The mean pixel value of each ROI was measured with Inveon Research Workshop software (Siemens Preclinical Solution). The value was then converted to the concentration of radioactivity in units of megabecquerels per milliliter. The image-derived tissue uptake, presented as percent injected dose per gram (%ID/g), was obtained with tissue radioactivity divided by injected dose assuming a tissue density of 1 g/mL.

Mouse genotyping. Mouse genotyping was performed on tail genomic DNA extracted using standard protocols. PCR amplifications were performed across the loxP site of the targeting construct, as well as across the *Mmut* cDNA to detect the INS-Alb-*Mmut* transgene. Primers used were: 5'-loxP site 5'-CCATTCTGGGAAGGCTTCTA-3' and 3'-loxP site 5'-TGCACAGAGTGCTAGTTTCCA-3'. For the murine Mmut cDNA : Forward: 5'-CATGTTGAGAGCTAAGAATC-3' and Reverse: 5'-TAGAAGTTCATTCCAATCCC-3'.

Diet and bezafibrate therapy. A 70% casein chow was fed *ad libitum* to the mice (TD.06723, Harlan Laboratories, Madison, WI) for 2 months. For the bezafibrate treatment study, mice were placed on a diet containing 70% casein and 0.5% bezafibrate (TD.10859 Custom Diet, Harlan Laboratories). The nutrient

information of the chow was: protein 60.9% by weight protein, 18.2% carbohydrate and 6.2% fat, providing 3.7kcal/gr (65.4%kcal from protein, 19.6 from carbohydrates and 15% from fat).

Clinical Chemistry screen. Terminal blood collections were obtained retro-orbitally from mice using heparinized glass capillary tubes (Drummond Scientific, Broomall, PA) following intraperitoneal injection of pentobarbital (5mg/mL, dose of 0.2-0.3 mL/10g body weight). The samples were centrifuged (4^0C, 10 min, 10,000 rpm), the plasma removed, and stored at -80C in a screw-top tube for later analysis. Methylmalonic acid was analyzed by gas chromatography–mass spectrometry with stable isotopic internal calibration, as previously described (6). Free fatty acids (Roche Diagnostics Gmbh, Mannheim, Germany), triglycerides (Pointe Scientific Inc., Canton, MI), cholesterol (Thermo Scientific, Middletown, VA) were measured by calorimetric assays.

Quantitative real-time PCR analysis. Total RNA from frozen tissue was extracted using the RNeasy Mini Kit (74104; Quiagen, Valencia, CA). DNase digestion was performed using DNA-free (AM1906; Ambion, Austin, TX) and 2μg of RNA was reverse transcribed using the High-Capacity cDNA Kit (4368814; Applied Biosystems, Foster City, CA). TaqMan gene expression assays were performed in triplicate according to the manufacturer's instructions using the Fast Universal PCR Master Mix (4352042, Applied Biosystems) and the Applied Biosystems 7500 Fast Real-Time PCR System. TaqMan probes specific to murine *PpargC1α* (Mm01208835_m1), *Ucp1* (Mm01244861_m1), *Dio2* (Mm00515664_m1), *Lcn2* (Mm01324470_m1) were used and mRNA expression was normalized to housekeeping *Gapdh* (Mm99999915_g1) (Applied Biosystems, Foster City, CA). Quantification of relative gene expression was calculated using the $2^{-\Delta\Delta CT}$ (comparative threshold) method.

Western analysis. Murine white and brown adipose tissues (WAT, BAT) were harvested from *Mmut* $^{-/-}$;Tg$^{INS\text{-}Alb\text{-}Mmut}$ and wildtype C57BL/6 mice with and without exposure to cold stress. Human white fat subcutaneous fat was obtained from MMA (index case subcutaneous fat from anterior upper left chest wall, during a port removal surgery, and a 34 year old mu0 female patient during a postmortem exam. Control human fat tissues

were obtained from a 26 and 29year old control female subjects, during postmortem examinations at the NIH Clinical Research Center. Tissues were homogenized using sterile homogenizer tubes and pestles on ice in T-PER (ThermoFisher Scientific) supplemented with fresh protease inhibitors (cOmplete Protease Inhibitor Cocktail, EDTA-free Sigma). Homogenates were centrifuged at 16,000 RCF for 10-15 minutes. Supernatant was collected and measured for protein content by Braford assay. Samples were then denatured in 5x SDS loading buffer and run on SDS-PAGE gels. Resulting Western blots were immunoblotted with one or more of the indicated antibodies: anti-β-actin (Proteintech, 66009-1-Ig), anti-propionyl (PTM Biolabs, PTM-203), anti-acetyl (PTM Biolabs, PTM-101), anti-succinyl (PTM Biolabs, PTM-401), anti-methylmalonyl (AP42053), anti-SIRT1 (Proteintech, 13161-1-AP), anti-SIRT5 (Proteintech, 15122-1-AP), anti-MFN2 (Proteintech, 67487-1-Ig), anti-UCP1 (Proteintech, 23673-1-AP), anti-PGC1α (Proteintech, 66369-1-Ig), anti-CREB1 (Proteintech, 67927-1-Ig), anti-DIO2 (Proteintech, 66813-1-Ig), and anti-FGF21 (abcam, EPR8314).

Histology, immunohistochemistry and electron microscopy. Tissues were fixed in 10% formalin, embedded in paraffin, sectioned, stained with hematoxylin and eosin following standard procedures (Histoserv), and examined by light microscopy. Sections of white fat, inguinal or subcutaneous were stained for UCP1 (ab-23841; Abcam) by immunohistochemistry, following the manufacturers' instructions [Ready-to-Use Vectastain Universal ABC Kit (Vector Labs)]. Tissue slides were analyzed with an Olympus microscope at a 200× magnification. Transmission electron microscopy was performed on tissues fixed at 4^0C in 2% glutaraldehyde in 0.1M cacodylate buffer (pH 7.4). The tissues were postfixed in 2% OsO_4 for 2h, washed again with 0.1M cacodylate buffer three times, subsequently washed with water and placed in 1% uranyl acetate for 1h. The tissues were serially dehydrated in ethanol and propylene oxide and embedded in EMBed 812 resin (Electron Microscopy Sciences, Hatfield, PA, USA). Thin sections, 80 nm thick, were obtained by utilizing an ultramicrotome (Leica, Deerfield, IL, USA), placed onto 300 mesh copper grids and stained with saturated uranyl acetate in 50% methanol and then with lead citrate. The grids were viewed in the JEM-1200EXII electron microscope (JEOL Ltd, Tokyo, Japan) at 80 kV and images were recorded on the XR611M, mid mounted, 10.5Mpixel, CCD camera (Advanced Microscopy Techniques Corp, Danvers, MA, USA).

SUPPLEMENTAL TABLES

Supplemental Table 1: Appendicular segment landmarks used for DXA analysis.

Segment	Landmarks	Coefficient of Variation (CV) MMA	Coefficient of Variation (CV) Controls
Upper arm (R1)	Glenohumeral joint between the head of the humerus and the glenoid fossa to humeroulnar joint between the outer and inner epicondyle.	3.1 ± 2.4%	2.5 ± 1.7%
Lower arm (R2)	Humeroulnar joint to hand.	2.6 ± 1.6%	3.2 ± 2.7%
Upper leg (R3)	Hip joint, line between the femoral neck and ischial tuberosity to the femorotibial joint.	3.2 ± 2.4%	3.4 ± 2.2%
Lower leg (R4)	Knee joint to foot	1.5 ± 3.1%	0.9 ± 1.6%

Supplemental Table 2. Pathway-enrichment analysis of significantly expressed serum proteomic markers between MMA patients and matched obese controls.

Canonical pathway	P-value	Proteins	Name	Fold Change (>1.5)	FDR (q value)
Osteoarthritis pathway	1.31E-31	MMP13	Matrix metallopeptidase 13	-2.322	1.02E-06
		TLR2	Toll like receptor 2	-1.894	1.42E-06
		GDF2	Growth differentiation factor 2	-1.671	1.74E-06
		IL1B	Interleukin 1 beta	-2.119	2.34E-06
		VEGDF	Vascular endothelial growth factor D	-1.579	6.39E-05
		CASP3	Caspase 3	1.86	1.53E-04
		HIF1A	Hypoxia inducible factor 1 subunit alpha	-1.774	1.74E-04
		SPHK1	Sphingosine kindase 1	1.721	3.80E-04
		DKK1	Dickkopf WNT signaling pathway inhibitor 1	-1.548	9.09E-04
		FN1	Fibronectin 1	-2.683	4.26E-03
Role of Macrophages, Fibroblasts and Endothelial cells in rheumatoids arthritis	1.67E-30	IL7	Interleukin 7	-1.699	1.05E-06
		JAK2	Janus kinase 2	2.401	1.46E-06
		PRSS2	Serine protease 2	2.011	1.57E-05
		PRKCB	Proteine kinase C beta	2.475	3.30E-05
		PRKCA	Proteine kinase C alpha	2.274	8.26E-05
		GDF15	Growth differentiation factor 15	1.604	9.51E-05
		MYC	Myc proto-oncogene	-1.573	1.53E-04
		GRB2	Growth factor receptor bound protein 2	1.945	1.94E-04

		MAPK3	Mitogen-activated protein kinase 3	1.859	5.91E-04
		PDGFB	Platelet derived growth factor subunit B	-1.814	1.51E-03
		PDGFA	Platelet derived growth factor subunit A	-1.802	1.66E-03
		STAT3	Signal transducer and activator of transcription 3	1.544	2.24E-03
Acute Phase Response signaling	6.16E-22	ITIH4	Inter-alpha-trypsin inhibitor heavy chain 4	2.817	2.36E-06
		ALB	Albumin	-1.772	2.89E-06
		C1R	Complement C1r	-1.741	3.32E-06
		PLG	Plasminogen	-1.747	1.99E-05
		C4A	Complement C4B	-2.406	2.70E-05
		IL1A	Interleukin 1 alpha	-1.630	3.74E-05
		PDPK1	3-phosphoinositide dependent protein kinase 1	1.621	1.50E-02
Neuroinflammation signaling pathway	1.46E-20	PLA2G2E	Phospholipase A2 group IIE	-1.779	1.02E-06
		BDNF	Brain derived neurotrophic factor	-2.254	1.57E-05
		MAPT	Microtubuie associated protein tau	-1.847	1.61E-05
		APP	Amyloid beta precursor protein	-1.943	3.85E-04
		SNCA	Synuclein alpha	1.922	3.24E-03
Coagulation system	4.52E-15	TFPI	Tissue factor pathway inhibitor	-1.543	7.26E-08
		F2	Coagulation factor II, thrombin	-5.115	7.26E-08
		PF4	Platelet factor 4	-6.688	4.62E-07
		SERPINA5	Serpin family A member 5	8.611	1.42E-06
		FGG	Fibrinogen gamma chain	6.073	2.02E-06
		FGB	Fibrinogen beta chain	5.346	3.32E-06
		FGA	Fibrinogen alpha chain	7.539	4.60E-06
		PLG	Plasminogen	-1.747	1.99E-05
		F5	Coagulation factor V	1.506	3.51E-05
		SERPINE1	Serpin family E member 1	-1.911	1.12E-04
		KLK3	Kallikrein related peptidase 3	-1.772	1.37E-03

Since the SOMASCAN platform did not include FGF21, we measured GDF15, that is closely correlated with FGF21 in our MMA patient cohort (7) similar to observations in various mitochondrial disorders(8). Both markers were significantly elevated in the subset of MMA patients included in the current report compared to matched controls (P<0.0001 for both, Supplemental Figure 6A) (r=0.541, P=0.0052, R^2=0.293). In addition to the dysregulation of several inflammatory pathways, significant differences were observed in a number of complement factors and members of the coagulation cascade, which will require further validation and exploration in the MMA patient cohort. Although not typically associated with a hypercoagulable state, there

have been reports of cases with venous thrombosis, especially in the perioperative setting, especially after a liver transplantation procedure .

SI REFERENCES:

1. Worgan LC, Niles K, Tirone JC, Hofmann A, Verner A, Sammak A, et al. Spectrum of mutations in mut methylmalonic acidemia and identification of a common Hispanic mutation and haplotype. *Hum Mutat.* 2006;27(1):31-43.
2. Rohloff JC, Gelinas AD, Jarvis TC, Ochsner UA, Schneider DJ, Gold L, et al. Nucleic Acid Ligands With Protein-like Side Chains: Modified Aptamers and Their Use as Diagnostic and Therapeutic Agents. *Mol Ther Nucleic Acids.* 2014;3(10):e201.
3. Candia J, Cheung F, Kotliarov Y, Fantoni G, Sellers B, Griesman T, et al. Assessment of Variability in the SOMAscan Assay. *Sci Rep.* 2017;7(1):14248.
4. Cheung F, Fantoni G, Conner M, Sellers BA, Kotliarov Y, Candia J, et al. Web Tool for Navigating and Plotting SomaLogic ADAT Files. *J Open Res Softw.* 2017;5.
5. Gavrilova O, Marcus-Samuels B, and Reitman ML. Lack of responses to a beta3-adrenergic agonist in lipoatrophic A-ZIP/F-1 mice. *Diabetes.* 2000;49(11):1910-6.
6. Marcell PD, Stabler SP, Podell ER, and Allen RH. Quantitation of methylmalonic acid and other dicarboxylic acids in normal serum and urine using capillary gas chromatography-mass spectrometry. *Anal Biochem.* 1985;150(1):58-66.
7. Manoli I, Pass AR, Harrington EA, Sloan JL, Gagne J, McCoy S, et al. 1-(13)C-propionate breath testing as a surrogate endpoint to assess efficacy of liver-directed therapies in methylmalonic acidemia (MMA). *Genet Med.* 2021;23(8):1522-33.
8. Riley LG, Nafisinia M, Menezes MJ, Nambiar R, Williams A, Barnes EH, et al. FGF21 outperforms GDF15 as a diagnostic biomarker of mitochondrial disease in children. *Mol Genet Metab.* 2022;135(1):63-71.

SUPPLEMENTAL FIGURES:

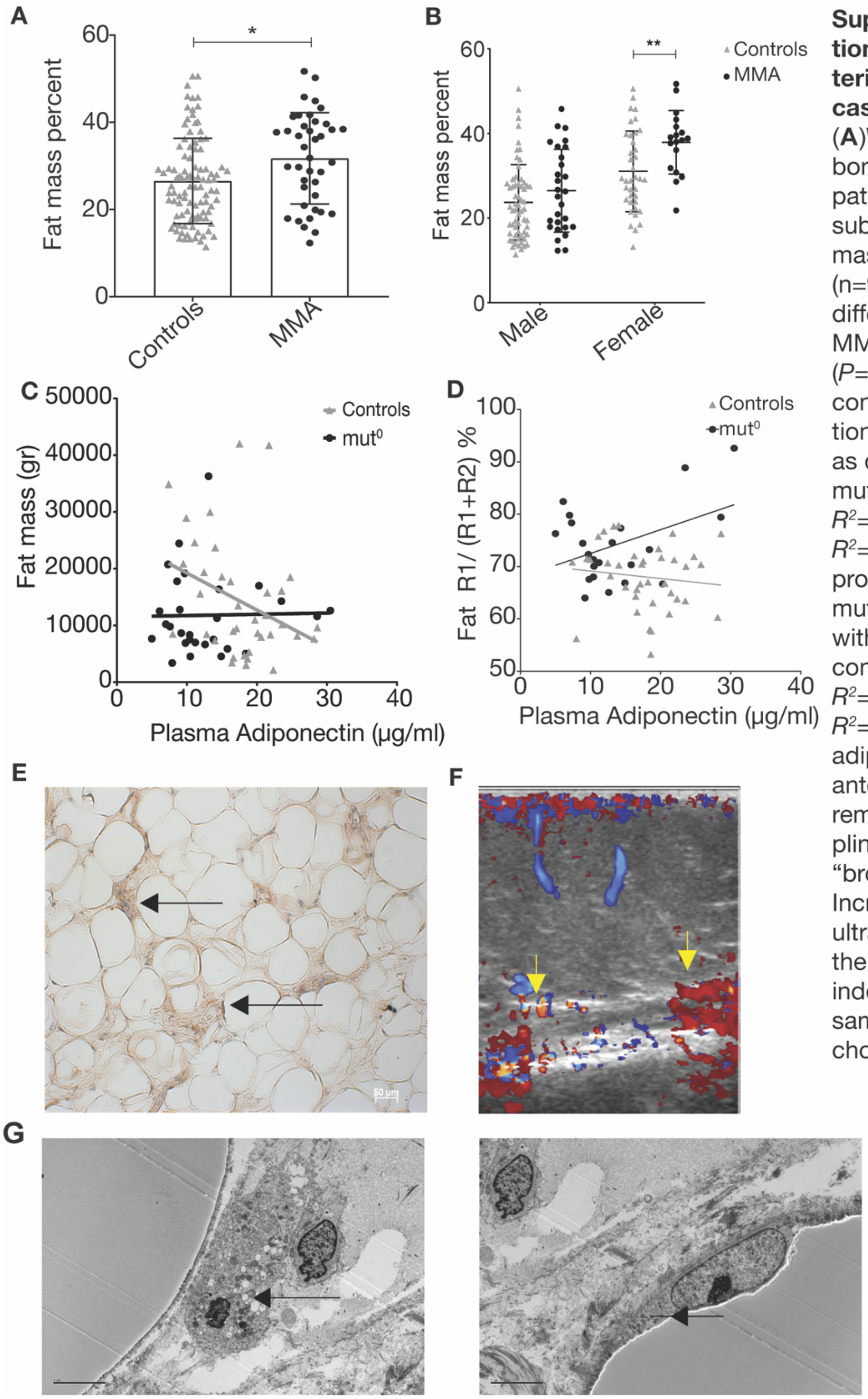

Supplemental Figure 1. Body composition analysis (DXA imaging) and characteristics of subcutaneous fat in the index case.
(**A**)Whole body composition analysis by bone densitometry (DXA) showed MMA patients (n=46) have a higher percent subtotal (total body minus head) body fat mass percent (FM%) compared to controls (n=99) (*P*=0.014, unpaired t-test). (**B**) The difference in FM% between control and MMA patients was significant in females (*P*=0.009), but not males. (**C**) Adiponectin concentrations showed a negative correlation with total fat mass in control subjects as opposed to no significant correlation in mut0 MMA subjects (r= -0.331, P=0.042, R^2=0.109 compared to r=0.019, P=0.91, R^2=0.0003, respectively). (**D**) The ratio of proximal/total FM% in upper extremities in mut0 MMA patients correlated positively with plasma adiponectin, but not in the control subjects (r=0.424, P=0.042, R^2=0.180 compared to r= -0.12, P=0.46, R^2=0.014 in the controls). (**E**) Subcutaneous adipose tissue obtained from the upper left anterior chest wall during a port-a-cath removal surgery stained positive for uncoupling protein-1, a marker of brown or "brown in white" (brite/beige) fat. (**F**) Increased vascularization was evident by ultrasound doppler studies in the area of the buffalo hump subcutaneous fat of the index case. (**G**) Electron microscopy of the same white fat sample showed rare mitochondria with abnormal ultrastructure.

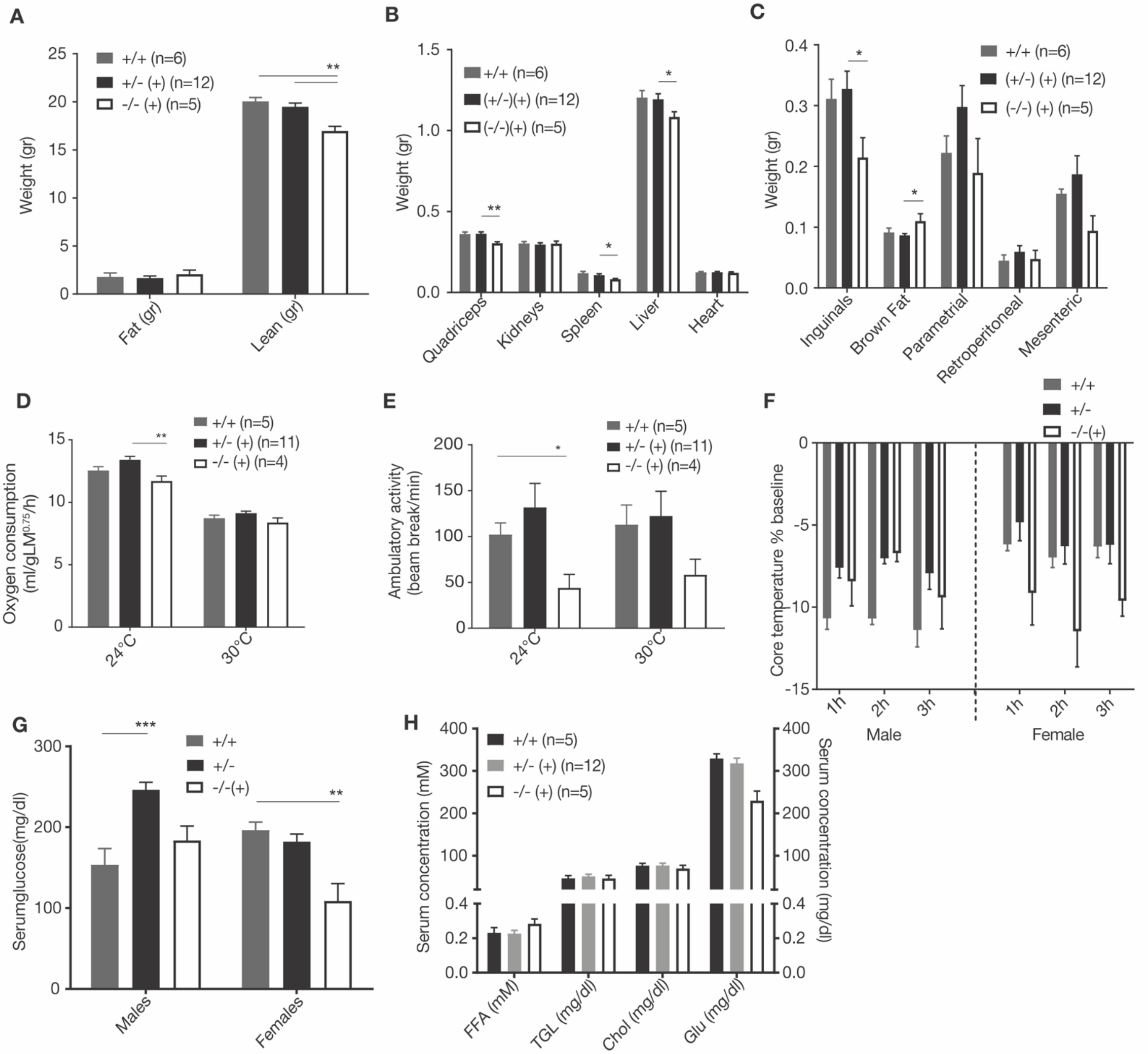

Supplemental Figure 2. Metabolic phenotyping of the liver-transgenic MMA mouse model, Mmut$^{-/-}$;Tg$^{INS\text{-}Alb\text{-}Mut}$. (A) Whole mouse DXA data are presented for wild type, heterozygote and transgenic *Mmut$^{-/-}$;Tg$^{INS\text{-}Alb\text{-}Mut}$* animals at baseline, on regular chow diet. Total lean mass in gr was decreased in the mutant mice (mean ± SEM, 16.968 ± 1.088 (*N*=5) compared to 20.04 ± 0.991 in wild type (*N*=6) and 19.48 ± 1.329 in heterozygote animals (*N*=12), adjusted *P*=0.001 between mutant and wild type group Tukey's multiple comparison, one-way ANOVA.) **(B)** Absolute weight in grams for the different organs between genotypes was lower in mutant animals for quadriceps (0.305 ± 0.008gr in mutant vs 0.361 ± 0.012 in wild type), spleen (0.082 ± 0.005gr vs 0.119 ± 0.011) and liver (1.084 ± 0.032 vs. 1.204 ± 0.042). (C) Individual fat pads were isolated and weighed for each genotype, showing lighter subcutaneous inguinal fat pads (0.215 ± 0.032gr in mutant vs 0.311 ± 0.032 in wild type) and slightly increased brown fat pads (0.110 ± 0.012 vs 0.091 ± 0.007) in mutant compared to wild type animals. **(D)** Oxygen consumption per gr of lean mass was lower in mutant transgenic mice (11.71 ± 0.8ml/g LM0.75/h vs 13.4 ± 0.9 in the heterozygote littermates), when mice were housed at 24oC room temperature, and associated with **(E)** lower motor ambulating activity (43.84 beam break/min in mutant mice compared to 102.0 in the wild type, *P*=0.04), as determined by infrared beam interruption. **(F)** Changes in core temperature were monitored closely during the 3-hour cold challenge. The largest decreases were observed in female mutant mice at all time points. **(G-H)** Serum glucose, free-fatty-acids, triglycerides, and cholesterol were measured in mice undergoing the metabolic chamber studies. Mutant female mice had lower serum glucose, while male heterozygote littermates had higher glucose compared to wild type control animals. No significant differences were noted in lipid profiles and FFA.

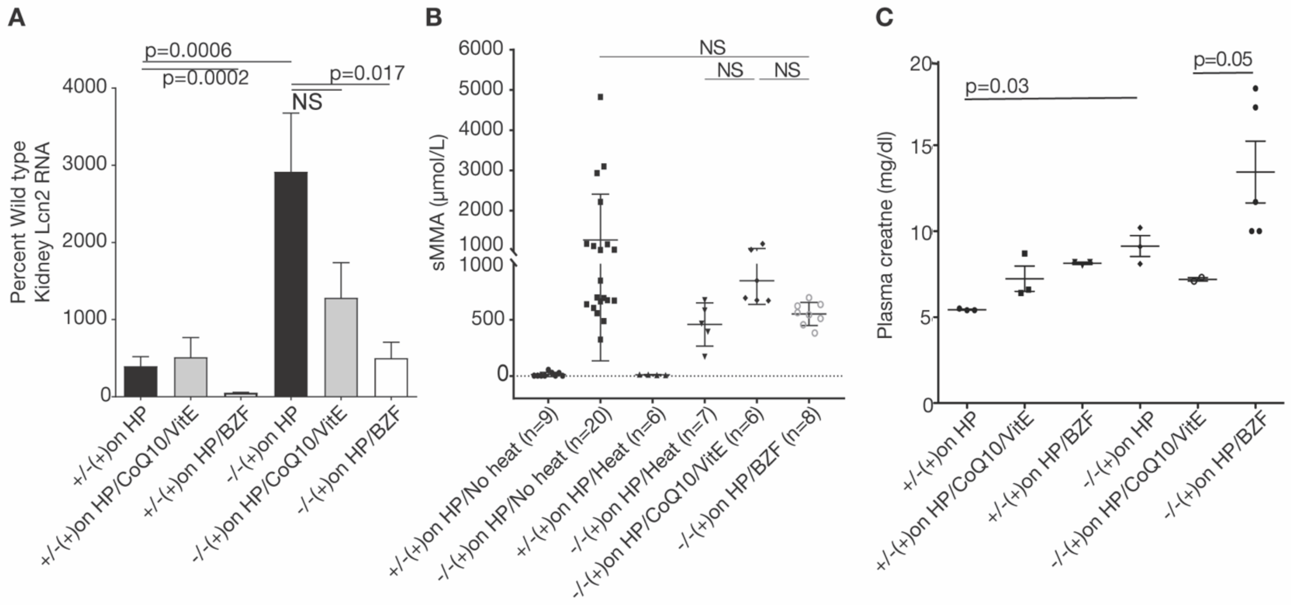

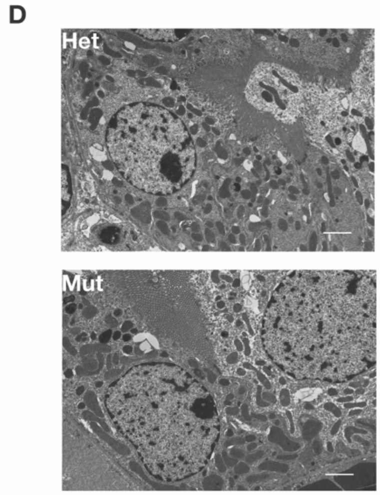

Supplemental Figure 3. Bezafibrate treatment effects on renal injury biomarkers and proximal tubule ultrastructure.
(A) Murine kidney tissues were analyzed by quantitative RT-PCR for expression of lipocalin-2 (NGAL, Lcn2), a marker of kidney injury in control and mutant mice (*Mmut*$^{-/-}$;*Tg*$^{INS\text{-}Alb\text{-}Mut}$) exposed to a high protein challenge for 2 months with or without additional treatments with antioxidants (coenzyme Q10/vitamin E) as described previously(36), or bezafibrates (BZF). Significant decrease in *Lcn2* mRNA expression was observed with BZF therapy as opposed to antioxidants. **(B)** Serum methylmalonic acid concentrations are shown for animals exposed to high protein diet with and without housing on heating pads compared to the two treatments of antioxidants (CoQ10/vitE) and BZF. Bezafibrate therapy showed low serum MMA levels despite a higher plasma creatinine shown in **(C)** Plasma creatinine measurements in mice exposed to high protein with or without the two treatment regimens (antioxidants vs BZF) showed higher creatinine values in bezafibrate-treated animals that was not accompanied by decreased glomerular filtration rate, as shown in Figure 4G or survival 4F. **(D)** Ultrastructure of mouse proximal tubules from mice exposed to high protein and bezafibrate therapy showed no apparent differences between mutant and heterozygote tissues in stark contrast to the previously published mitochondrial anomalies exacerbated by high protein in this murine model (36).

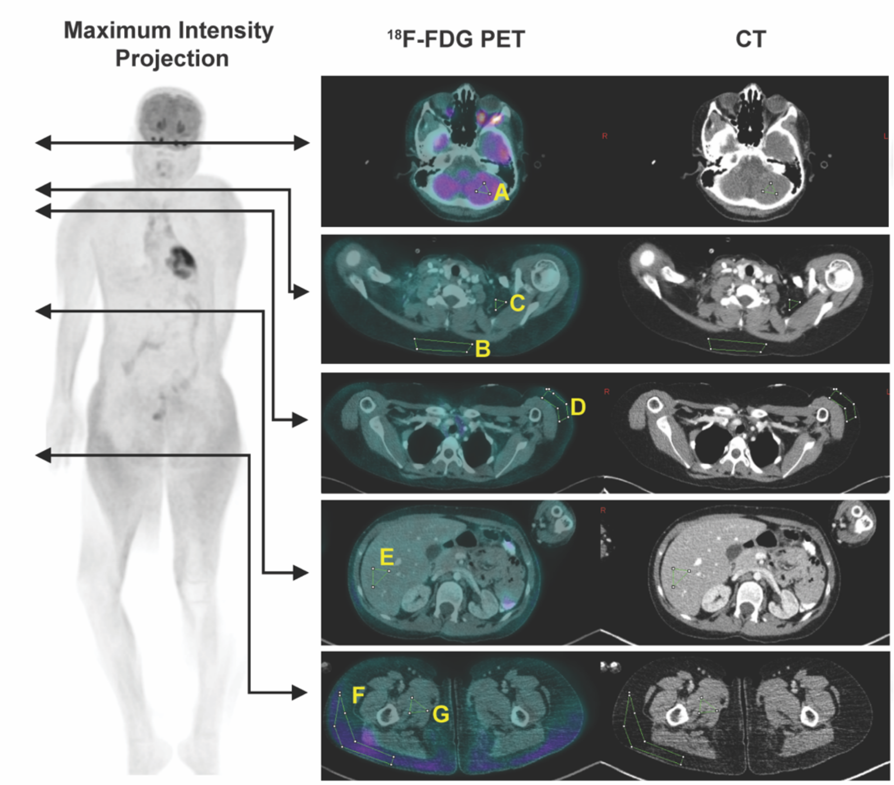

Label	Depot	SUV Max	SUV Mean
A	Cerebellum	3.52	2.23
B	Subcutaneous Adipose Tissue	0.77	0.56
C	Intermuscular Supraclavicular Adipose Tissue	0.99	0.88
D	Upper Arm Adipose	1.40	0.92
E	Liver	0.72	0.70
F	Leg Adipose	2.61	1.94
G	Quadricep	0.78	0.70

Supplemental Figure 4. Analysis of positron emission tomography study in a *mut⁰* MMA patient
Standardized uptake values (SUV) of [^{18}F]fluorodeoxyglucose are presented at different cross-sectional regions comparing uptake in solid organs (cerebellum, liver, muscle) and adipose tissue depots. Mean and max analysis of each region are provided in the adjacent table. Unusually high concentration of FDG uptake is observed in subcutaneous white adipose tissue of upper arm and leg (marked D and F respectively).

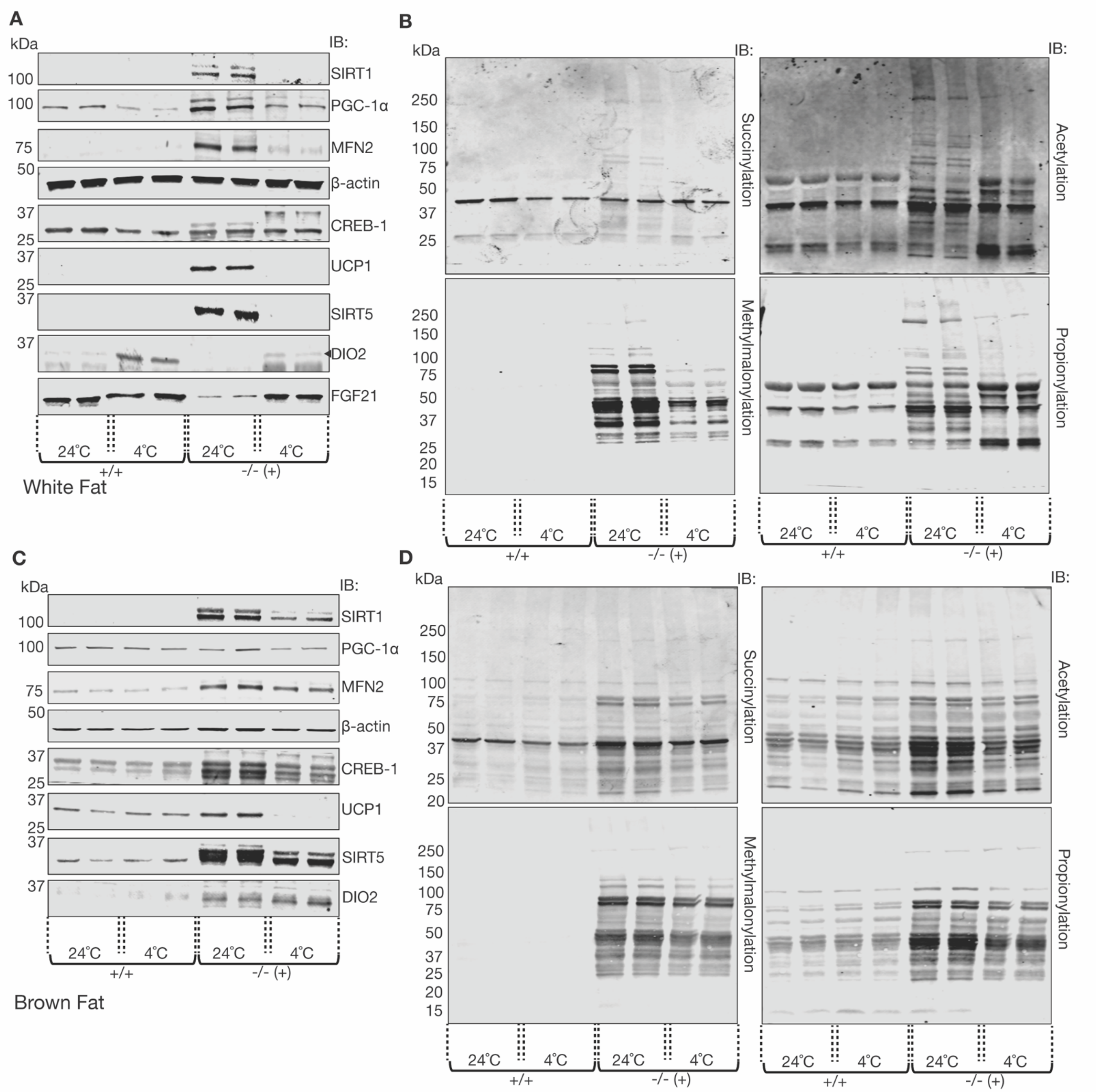

Supplemental Figure 5. Immunoblotting of white and brown adipose tissue in murine models. Protein expression in adipose tissue of mutant mice (*Mmut*$^{-/-}$;*Tg*$^{INS\text{-}Alb\text{-}Mut}$) and wild type littermates at baseline (24˚C) and after cold challenge (4˚C for 3 hours) is shown for markers of beige/brown fat and post-translational modifications. **(A)** Subcutaneous white fat tissue from the inguinal depot showed higher expression of PGC-1α, MFN2, SIRT1, CREB1 and phosphorylated CREB1, UCP1 and SIRT5, with lower levels of FGF21 at baseline room temperature (24˚C). Cold exposure induced expression of DIO2, and FGF21 in wild type and mutant mouse tissues, and likely the sumoylated CREB1 in mutant mouse tissues, while it decreased the expression of PGC-1α, MFN2, SIRT1, CREB1, UCP1 and SIRT5 in the mutant animals. **(B)** White fat tissue PTMs were all increased in mutant mice (*Mmut*$^{-/-}$;*Tg*$^{INS\text{-}Alb\text{-}Mut}$) tissues, especially methylmalonylation, which was absent from control animals. A slight decrease in methylmalonylation was observed after cold exposure, while propionyl and acetylation showed a different band pattern and intensity after cold exposure in mutant mice.**(C)** Brown fat of mutant mice showed higher expression of MFN2, SIRT1, CREB-1, and the mitochondrial proteins UCP1, DIO2 and SIRT5. Cold exposure caused a downregulation of UCP1, CREB1, MFN2, and SIRT5 only in the mutant animals. **(D)** Brown fat tissue PTMs were all increased in mutant mice (*Mmut*$^{-/-}$;*Tg*$^{INS\text{-}Alb\text{-}Mut}$) tissues with a slight decrease after cold exposure. Methylmalonylation was absent from control animals.

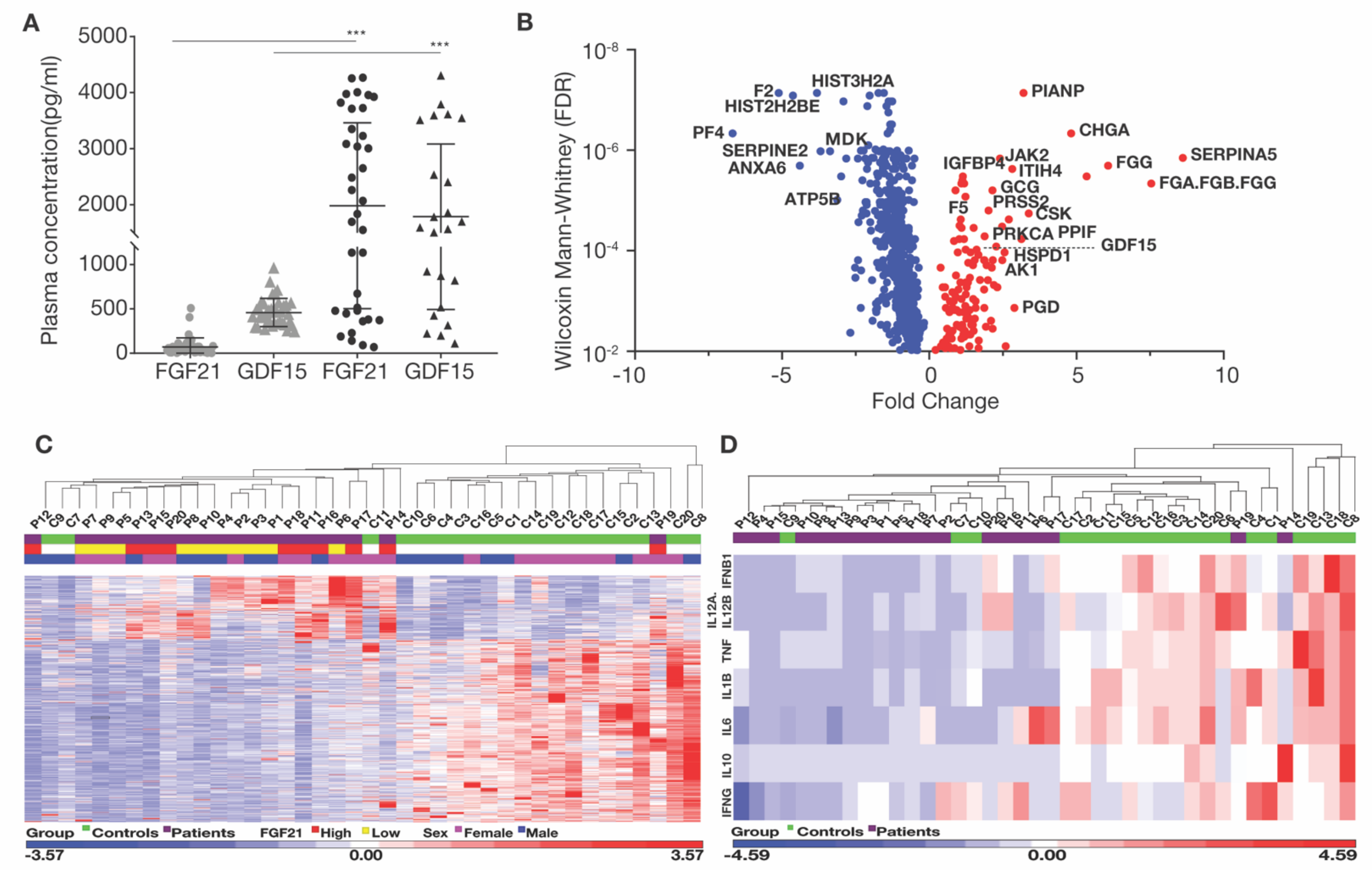

Supplemental Figure 6. A proteomic screen for markers associated with the significant elevations in FGF21 and GDF15 observed in MMA patients revealed a benign metabolic profile compared to controls. **(A)** Methylmalonic acidemia patients had significantly higher plasma concentrations of both FGF21 (1982 ±1480pg/ml vs. 71.47 ± 102.5 mean ± SD, *N*=39, *P*<0.0001, Mann-Whitney test) and GDF15 (1788 ± 1296 vs. 459 ± 159.3, *P*<0.0001) compared to matched control subjects. **(B)** Volcano plot of the differential expression of blood proteins from MMA and matched controls subjects (*N*=20 for each) measured with the SOMASCAN aptamer assay. The statistically most significant proteins are shown toward the top of the graph, while the proteins with the largest positive or negative fold-change are shown toward the upper right and left, respectively. GDF15 is amongst the significantly upregulated markers, as expected. **(C)** Hierarchical clustering of differentially expressed plasma proteins with adherence to stringent constraints (*P*<0.01, False Discovery Rate) after quantile normalization. Clustering was generated by unsupervised linkage. MMA patients clustered together except for one individual (P19). The abundance of each protein marker is shown in color, with red meaning overabundant proteins, white indicating unchanged and blue underabundant proteins compared to the reference mean (color bar scale at the bottom of the figure panel. Despite the extreme biochemical abnormalities and significantly elevated plasma FGF21 and GDF15 measured in MMA patients, the proteomic profile showed that several cytokine/inflammatory pathways were more abundant in the matched control obese subjects. **(D)** A subset of cytokines typically elevated in obesity and the metabolic syndrome (IFNγ, TNF, IL6, IL12, IL1B) is depicted showing overall lower circulating levels in the MMA cohort compared to the controls (Euclidean distance, complete agglomerative hierarchical clustering).
