# Supplementary material for: Lipodystrophy in methylmalonic acidemia associated with elevated FGF21 and abnormal methylmalonylation
Source: JCI Insight. 2024 Feb 22;9(4):e174097. doi: 10.1172/jci.insight.174097 (PMC10967474; doi:10.1172/jci.insight.174097)

For Figure 5 C (most blots were cut prior to staining in order to stain for multiple proteins) :

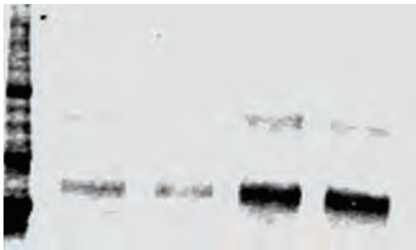

SIRT1

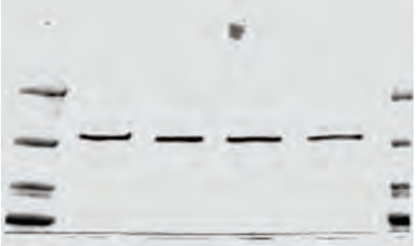

PGC-1α

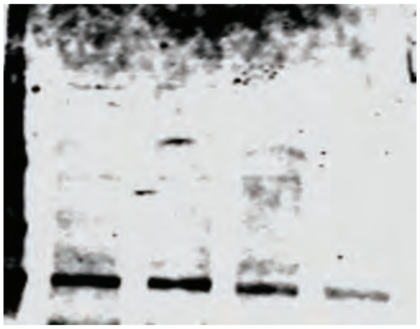

MFN2

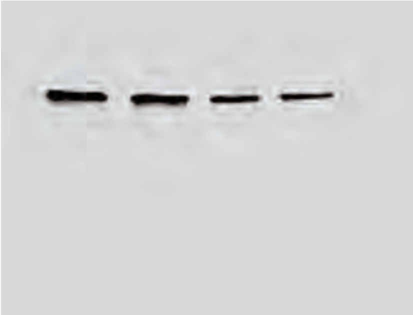

β-actin

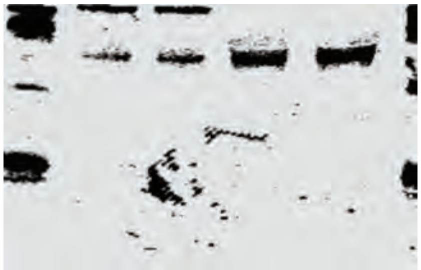

UCP1

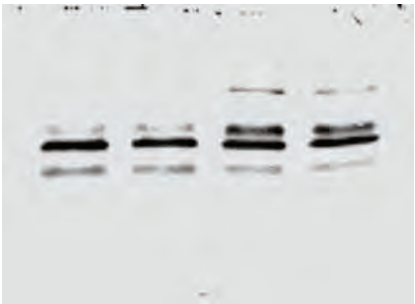

SIRT5

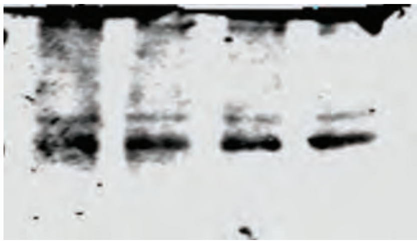

DIO2

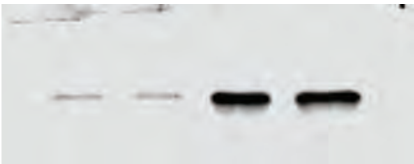

FGF21

For Figure 5 F (most blots were cut prior to staining in order to stain for multiple proteins) :

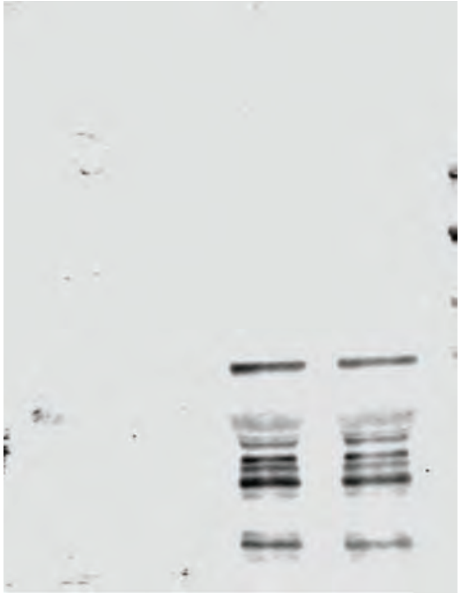

Methylmalonylation

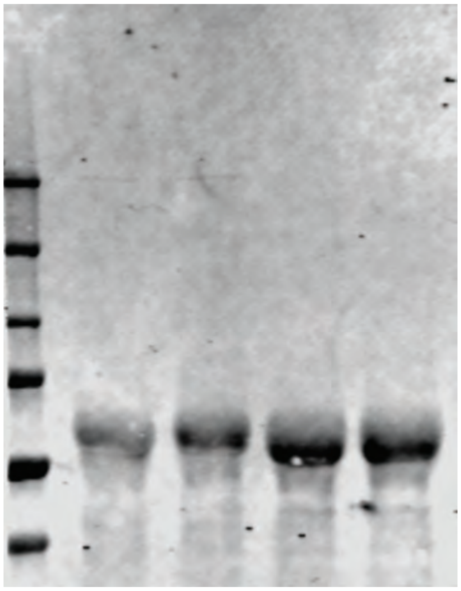

Acetylation

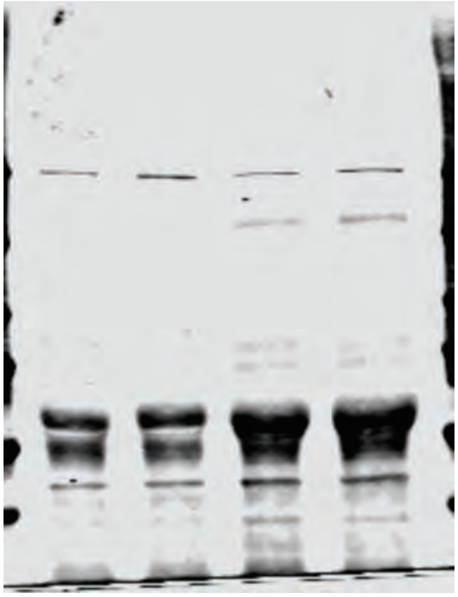

Propionylation

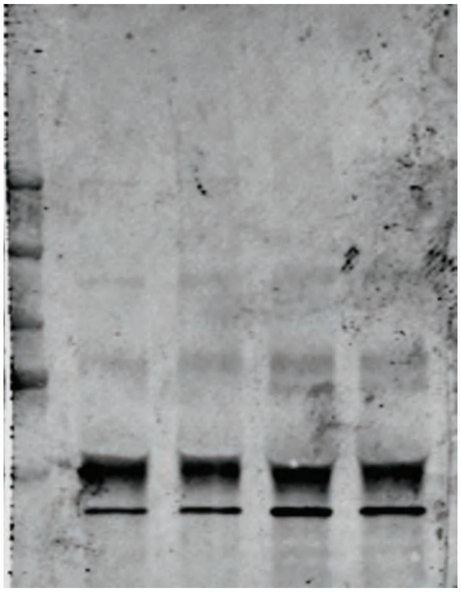

Succinylation

For Supplemental Figure 5 A (most blots were cut prior to staining in order to stain for multiple proteins) :

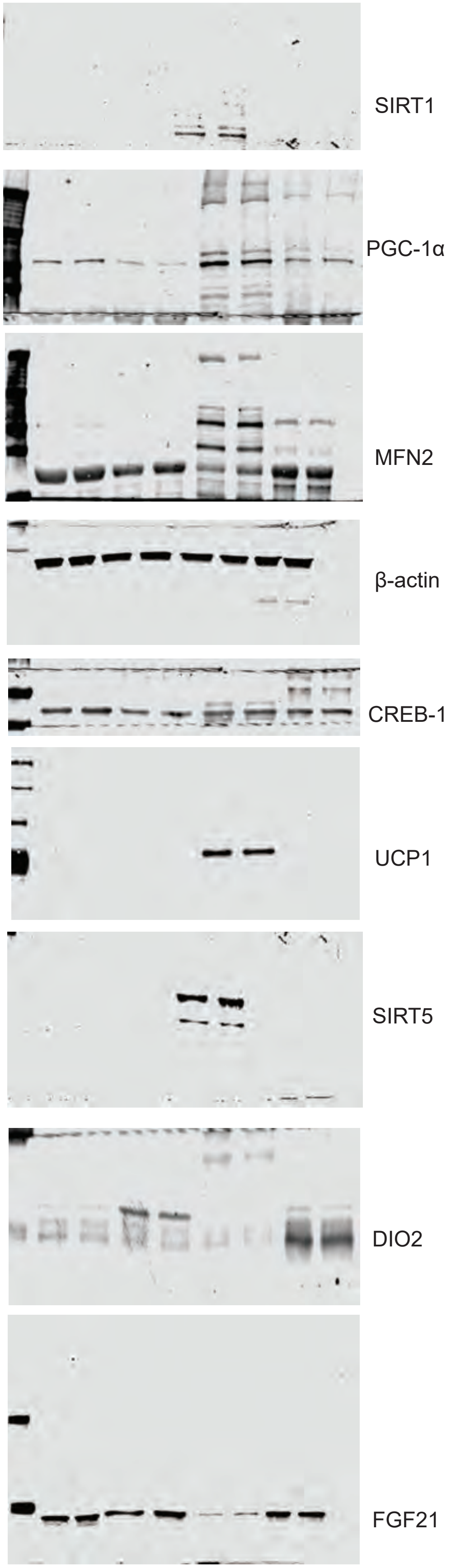

For Supplemental Figure 5 B (most blots were cut prior to staining in order to stain for multiple proteins) :

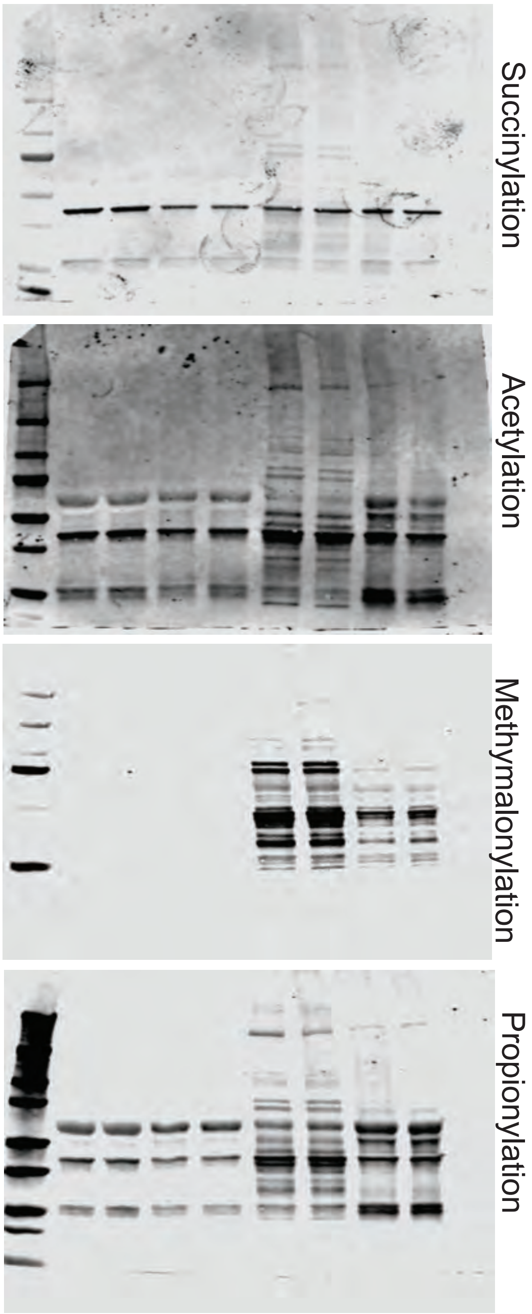

For Supplemental Figure 5 C (most blots were cut prior to staining in order to stain for multiple proteins) :

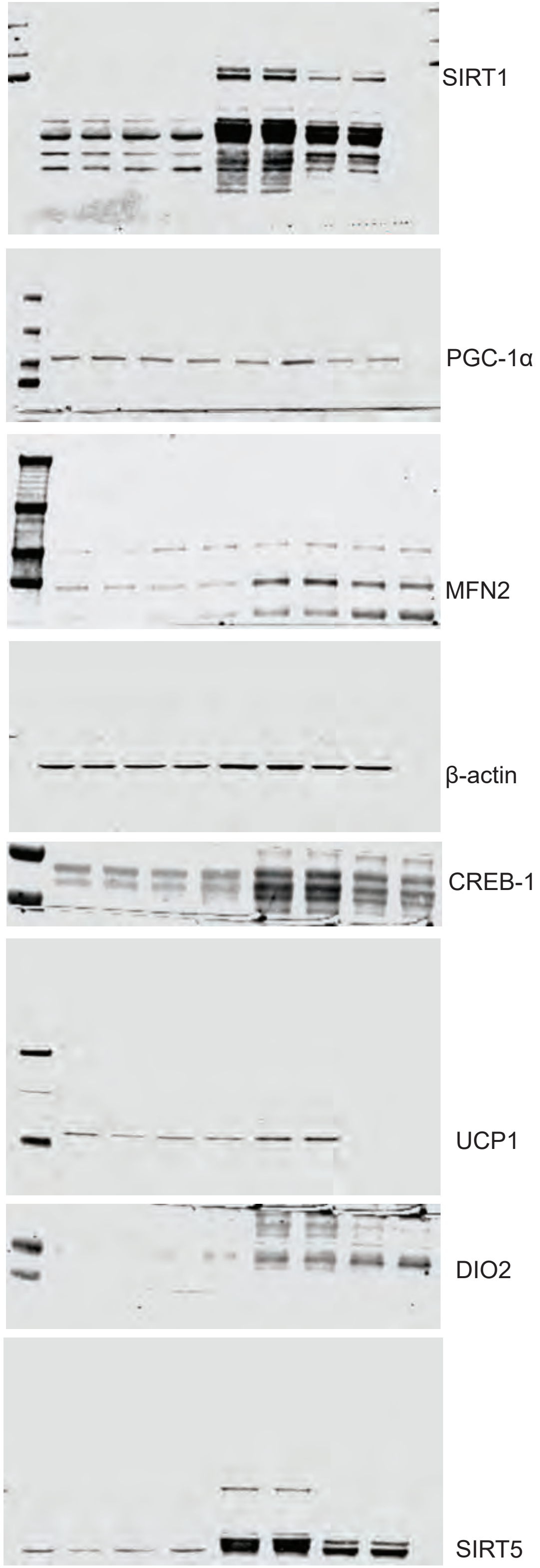

For Supplemental Figure 5 D (most blots were cut prior to staining in order to stain for multiple proteins) :

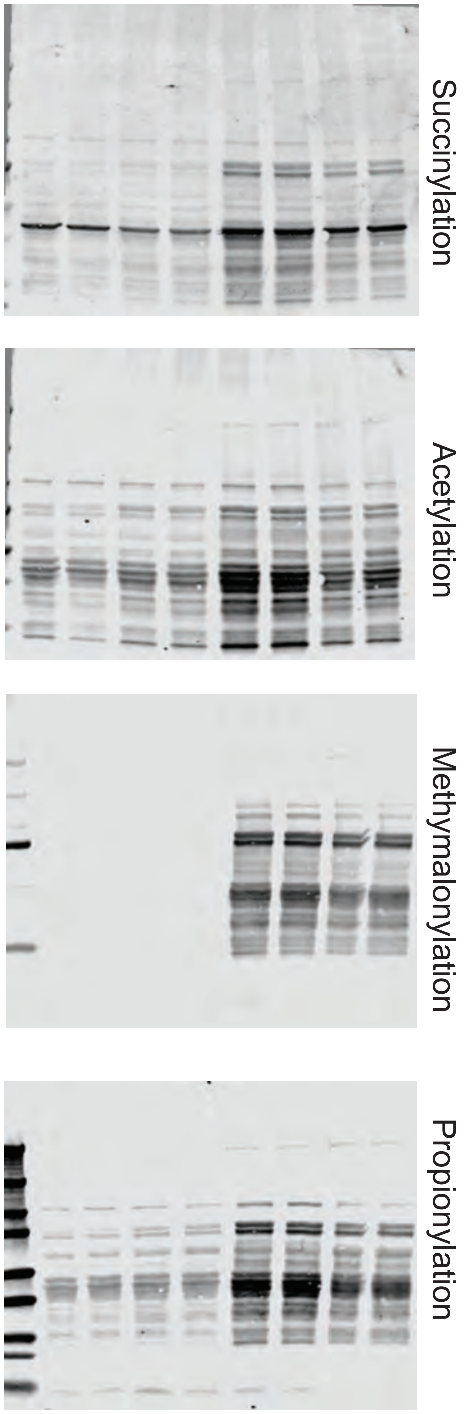

Supplement: Unedited blot and gel images [file jciinsight-9-174097-s147.pdf]
